# Supplementary material for: Host liver-derived extracellular vesicles deliver miR-142a-3p induces neutrophil extracellular traps via targeting WASL to block the development of Schistosoma japonicum
Source: Mol Ther. 2022 Mar 26;30(5):2092–107. doi: 10.1016/j.ymthe.2022.03.016 (PMC9092393; doi:10.1016/j.ymthe.2022.03.016)

YMTHE, Volume *30*

Supplemental Information

Host liver-derived extracellular vesicles deliver miR-142a-3p induces neutrophil extracellular traps via targeting WASL to block the development of *Schistosoma japonicum*

Lifu Wang, Zifeng Zhu, Yao Liao, Lichao Zhang, Zilong Yu, Ruibing Yang, Ji Wu, Zhongdao Wu, and Xi Sun

Supplemental Information

Host Liver-Derived Extracellular Vesicles Deliver miR-142a-3p Induces Neutrophil Extracellular Traps via Targeting WASL to Block the Development of *Schistosoma japonicum*

Lifu Wang, Zifeng Zhu, Yao Liao, Lichao Zhang, Zilong Yu, Ruibing Yang, Ji Wu, Zhongdao Wu, and Xi Sun

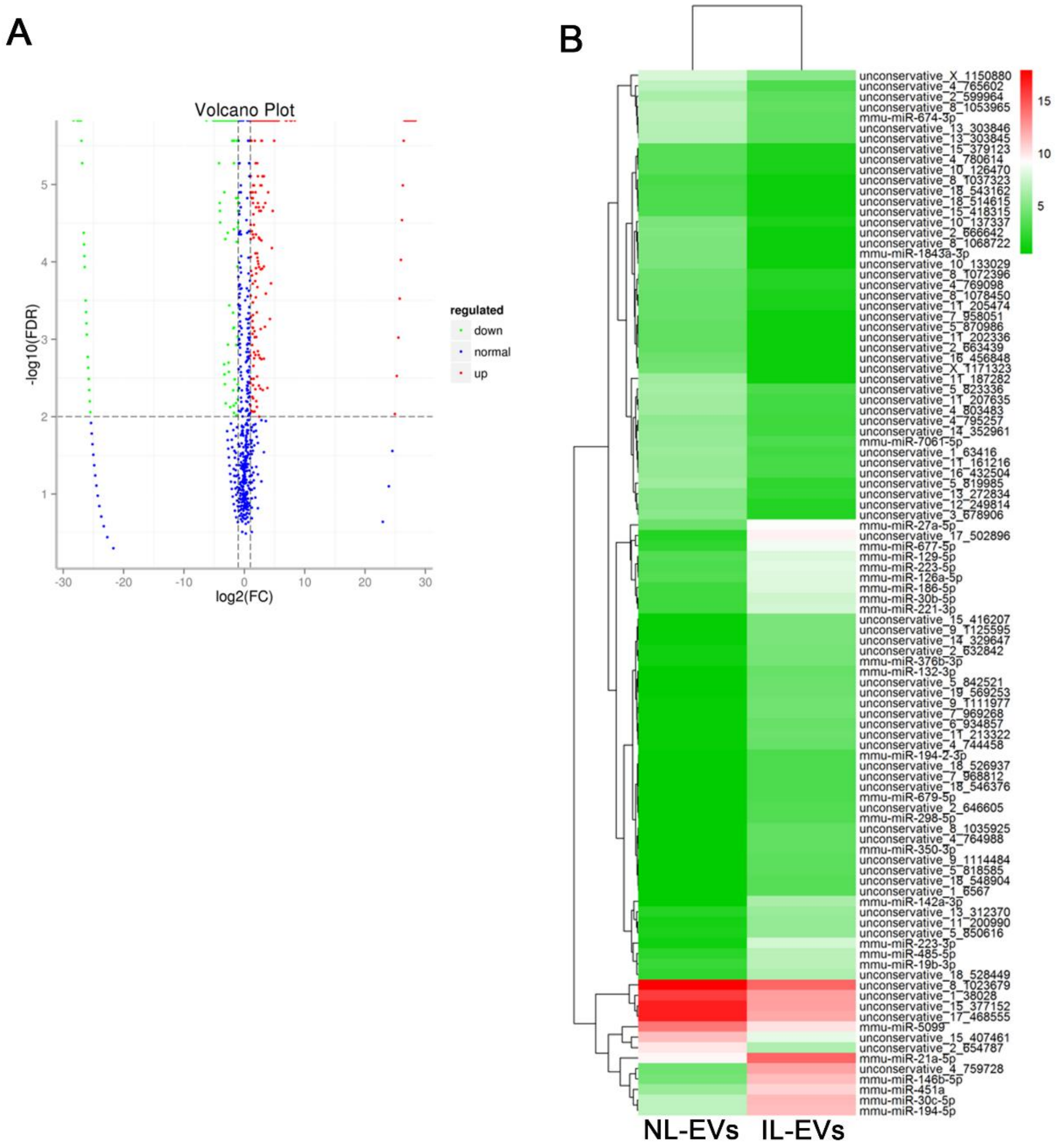

Figure S1. miRNAs of EVs derived from liver of *S. japonicum*–infected mice (IL-

EVs) and normal liver–derived EVs (IL-EVs) were sequenced and analyzed. (A) Volcano diagrams of miRNAs derived from the comparative gene expression analyses. Red dots represent up-regulated miRNAs, and green dots represent down-regulated miRNAs. **(B)** Heat map analysis shows differentially expressed miRNAs. Bright green=low expression; bright red=high expression.

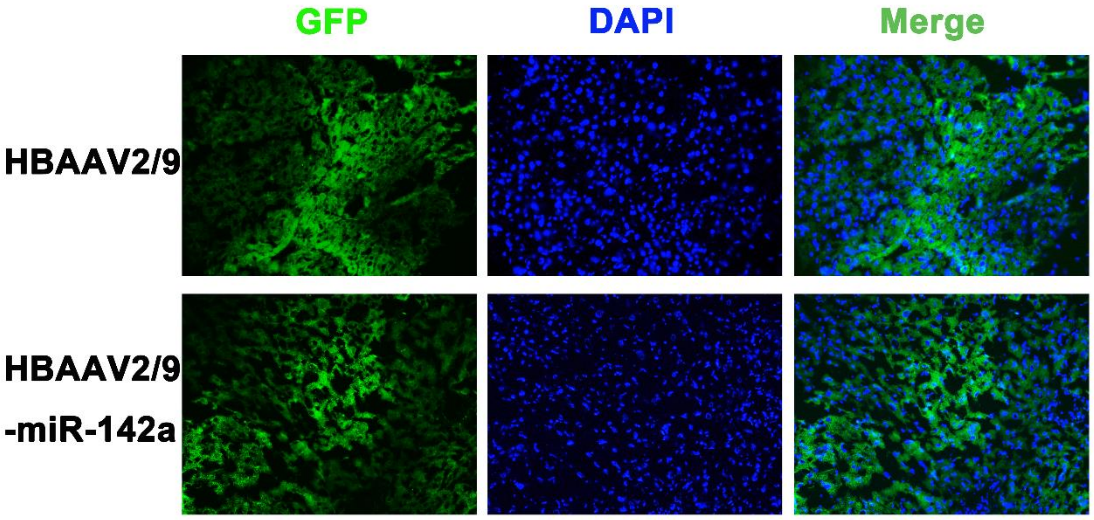

Figure S2. HBAAV2/9-miR-142a-3p was constructed and successfully transfected into mice (HBAAV2/9 expresses GFP). HBAAV2/9-miR-142a-3p colonization in the liver was analyzed by fluorescence microscopy.

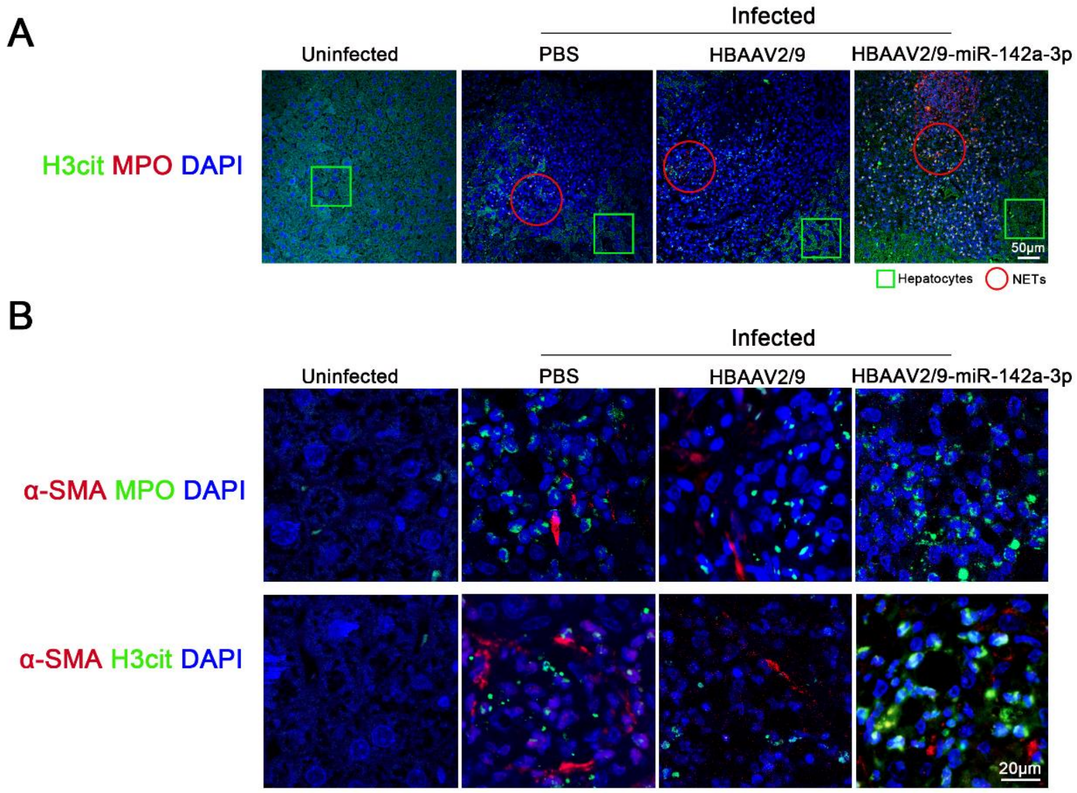

Figure S3. NETs were observed in the granulomas of *S. japonicum*. (A) Location of NETs in liver sections was observed based on H3cit and MPO co-localization. **(B)** Co-localization of H3cit, MPO, and α-SMA was observed by immunofluorescence.

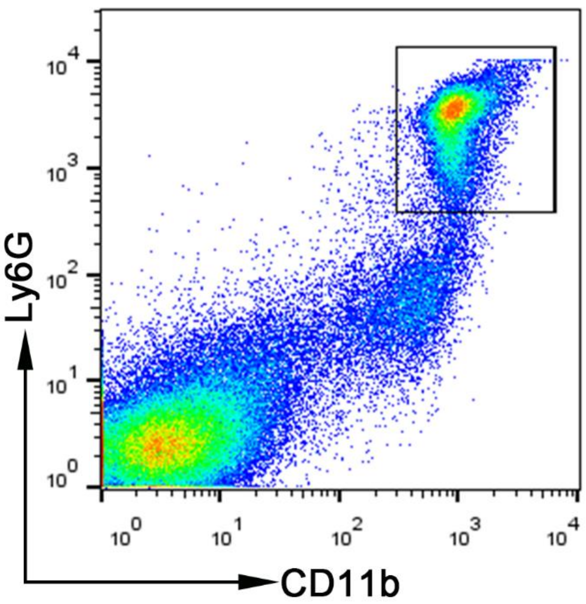

Figure S4. Neutrophils were isolated using Percoll density gradient centrifugation and flow cytometry.

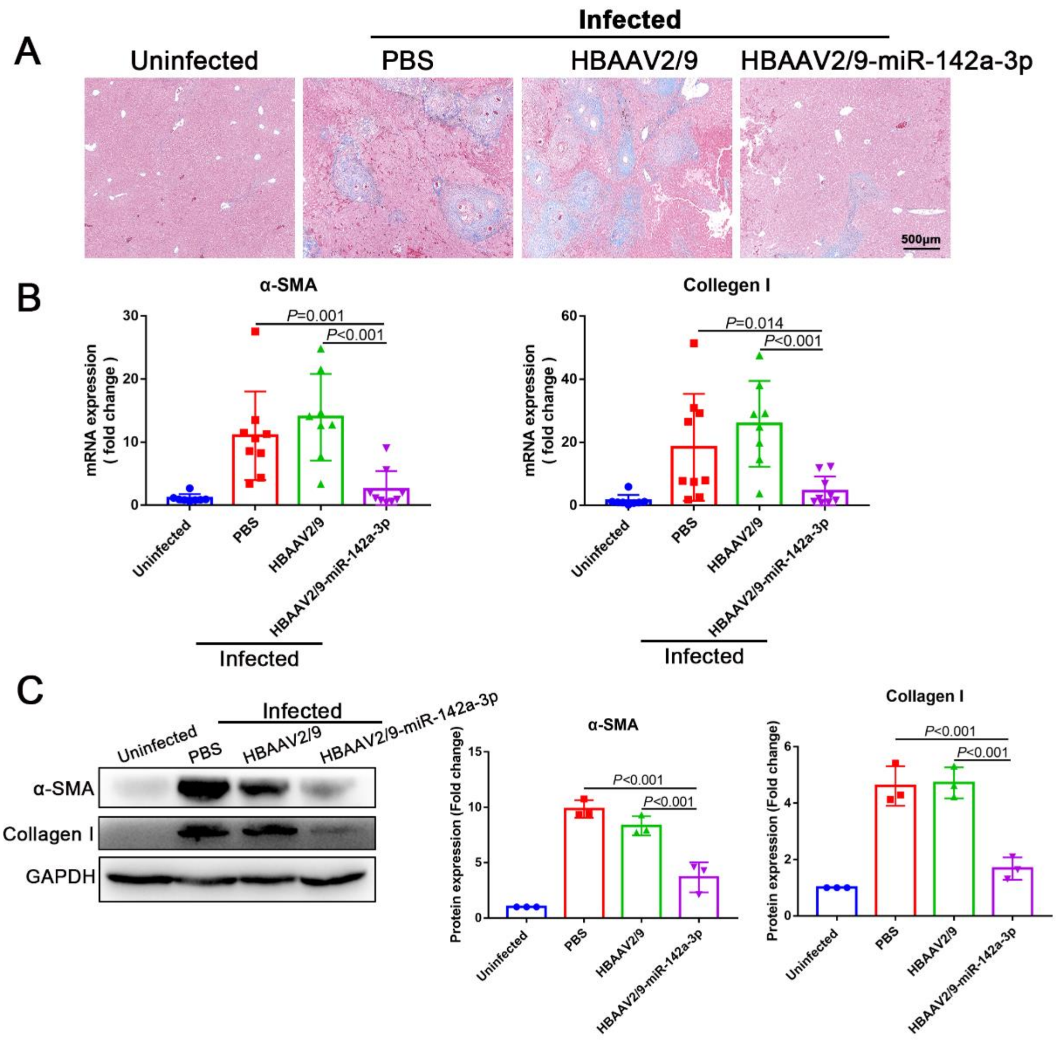

Figure S5. miR-142a-3p attenuated liver fibrosis in *S. japonicum* infection. (A) Fibrosis was observed in liver sections stained with Masson's trichrome. **(B)** Expression levels of α-SMA and collagen I in mouse liver were analyzed by qRT-PCR (n=8-9 mice per group). **(C)** Expression levels of α-SMA and collagen I in mouse liver were analyzed by Western blotting. Results are shown as mean ± SD (one-way ANOVA with Dunnett's multiple comparison test).

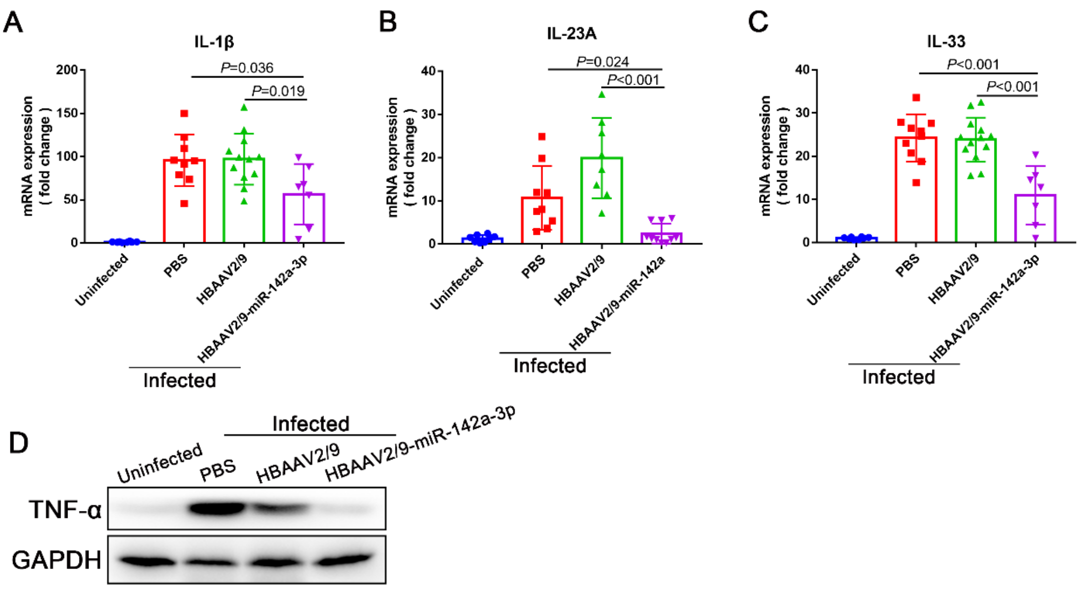

Figure S6. High expression of the inflammatory cytokines IL-1β **(A)**, IL-23A **(B)**, IL-33 **(C)**, and TNF-α **(D)** in liver tissues during *S. japonicum* infection decreased significantly after HBAAV2/9-miR-142a-3p treatment (n=7-12 mice per group).

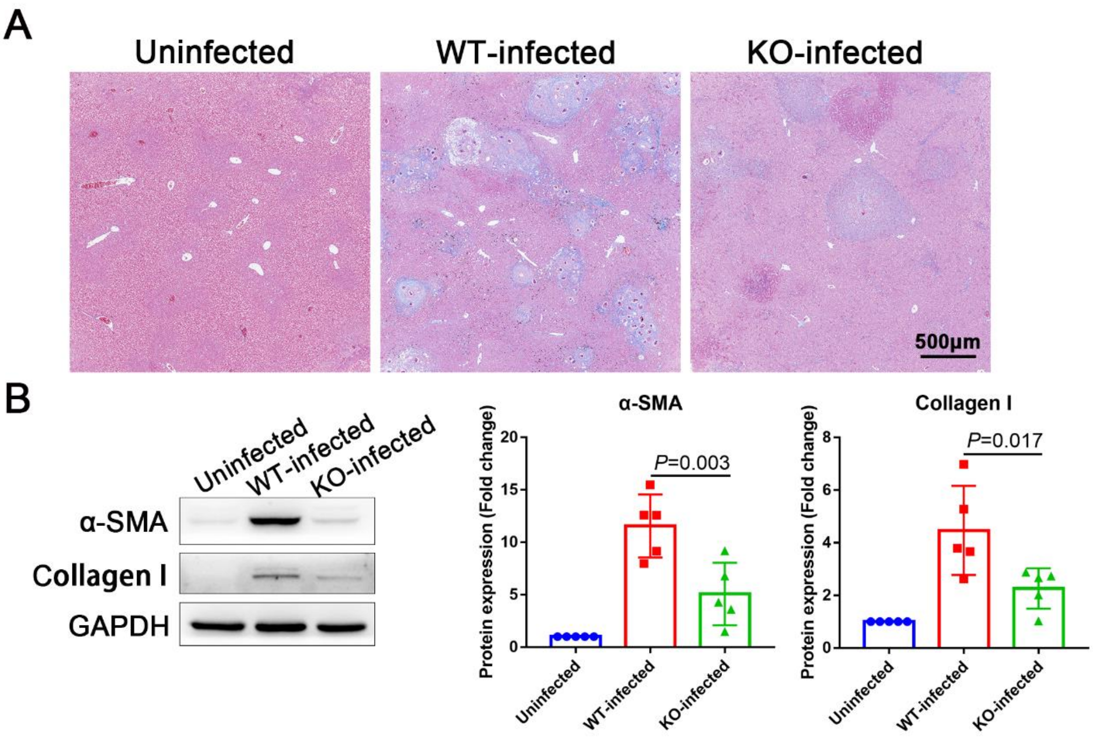

Figure S7. WASL-deletion attenuated liver fibrosis in *S. japonicum* infection. (A) Fibrosis was observed in liver sections stained with Masson's trichrome. **(B)**

Expression levels of α-SMA and collagen I in mouse liver were analyzed by Western blotting. Results are shown as mean ± SD (one-way ANOVA with Dunnett's multiple comparison test).

Table S1. Particle characteristics and yield of NL-EVs and IL-EVs

	NL-EVs	IL-EVs
Mice	n=10	n=10
Cell culture time of liver tissues	24 h	24 h
Cell density (cells/mL)	1×10^{6}	1×10^{6}
Particle concentration (particles/mL)	9.72×10^{10}	6.69×10^{10}
Protein concentration (mg/mL)	7.34	5.21
Total volume of EVs (in PBS)	300 µL	400 µL

Table S2. The top ten differentially-expressed miRNA (IL-EVs/NL-EVs).

NO.	miRNA	log2FC	regulated
1	mmu-miR-142a-3p	7.753542806	Up
2	mmu-miR-223-3p	7.615122837	Up
3	mmu-miR-677-5p	7.006138383	Up
4	mmu-miR-146b-5p	6.866236731	Up
5	mmu-miR-186-5p	5.572362953	Up
6	mmu-miR-132-3p	5.49367909	Up
7	mmu-miR-350-3p	5.333210545	Up

8	mmu-miR-485-5p	5.322722633	Up
9	mmu-miR-30b-5p	5.315091289	Up
10	mmu-miR-221-3p	5.176670648	Up

WASL gene knockout mice

Gene: ENSMUSG00000031165

Description: Wiskott-Aldrich syndrome [Source:MGI Symbol;Acc:MGI:105059]

Location: Chromosome X: 7,947,692-7,956,737 reverse strand.

Primers' sequences: "GGCCCTGGAGGACTTATTTC" and "AGCTCAGGGGGTCACTGATA"

PCR products: WT (811 bp) and KO (~200 bp)

PCR Reaction	
2×Taq Master Mix	7.5 µL
Forward primers (10 µM)	0.3 µL
Reverse primers (10 µM)	0.3 µL
Genomic DNA (20 ng/µL)	1.0 µL
Add H2O up to	15 µL

PCR Program		
	94 °C	5 min
35 cycles	94 °C	30 sec
	60 °C	30 sec
	72 °C	70 sec
	72 °C	10 min

PCR products: WT (811 bp) and KO (~200 bp)

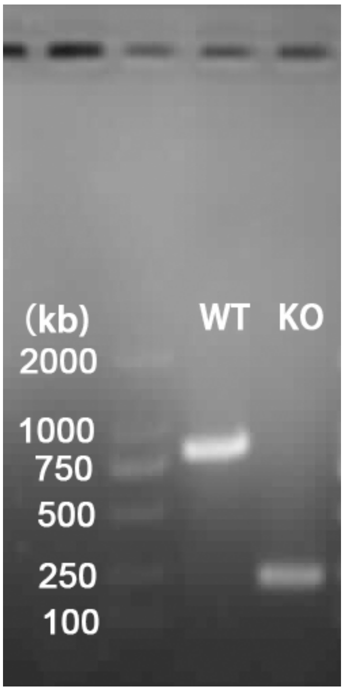
(kb)
WT
KO
2000
1000
750
500
250
100

Negative control for immunofluorescence

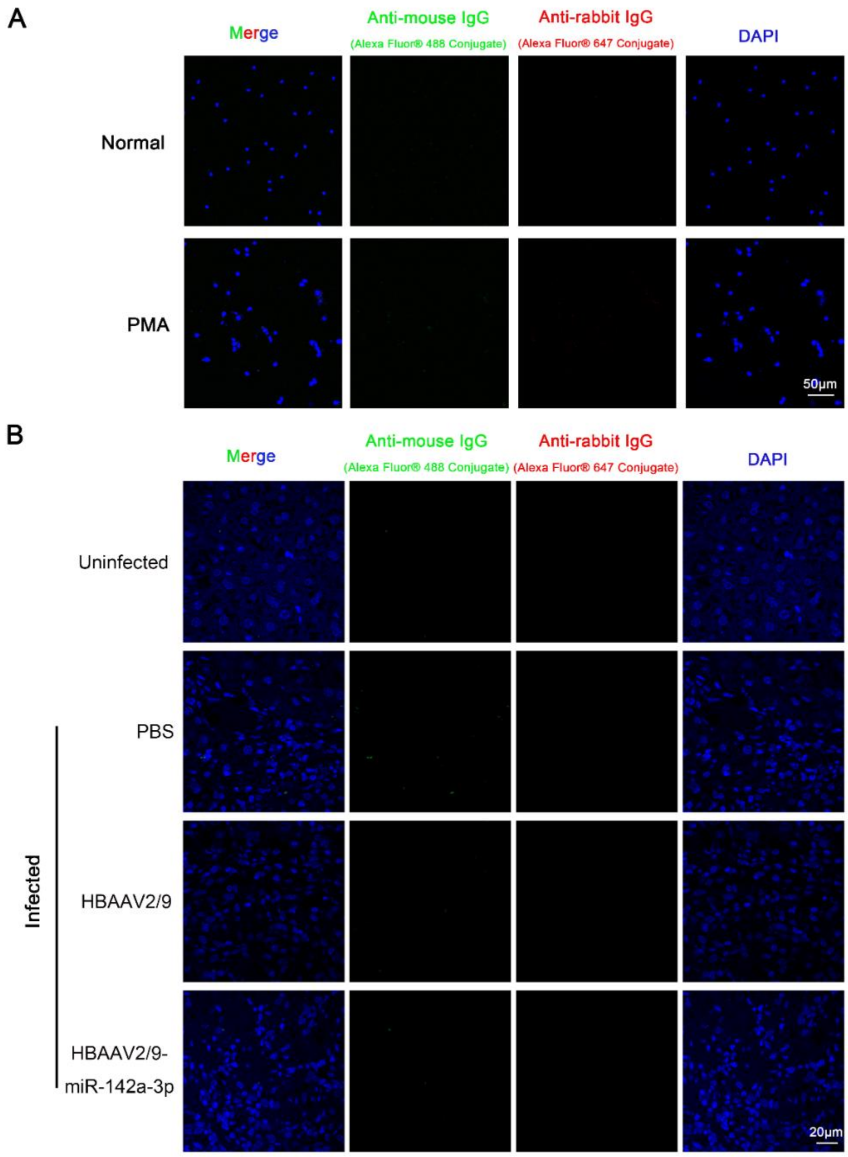

Negative control for immunofluorescence

Figure (A) Neutrophils were treated with PMA (500 nM, 4 h), and the non-specific staining effect of the secondary antibody was excluded by direct use of secondary antibody without adding primary antibody. **(B)** The non-specific staining effect of the secondary antibody on liver slices was excluded by direct use of secondary antibody without adding primary antibody.

Uncropped western blotting

Figure 1B

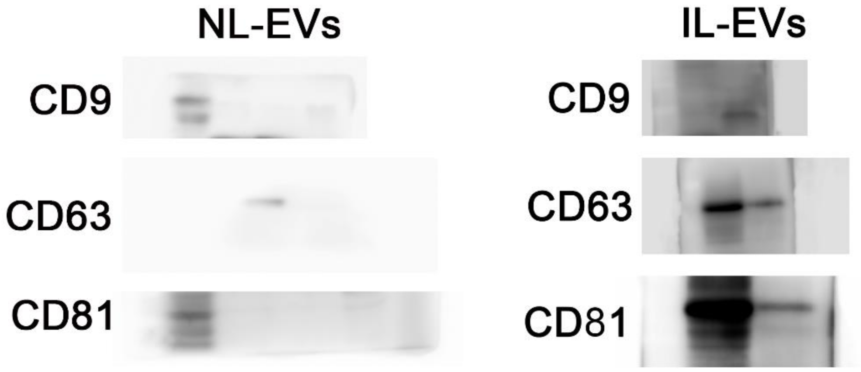

Figure 5C

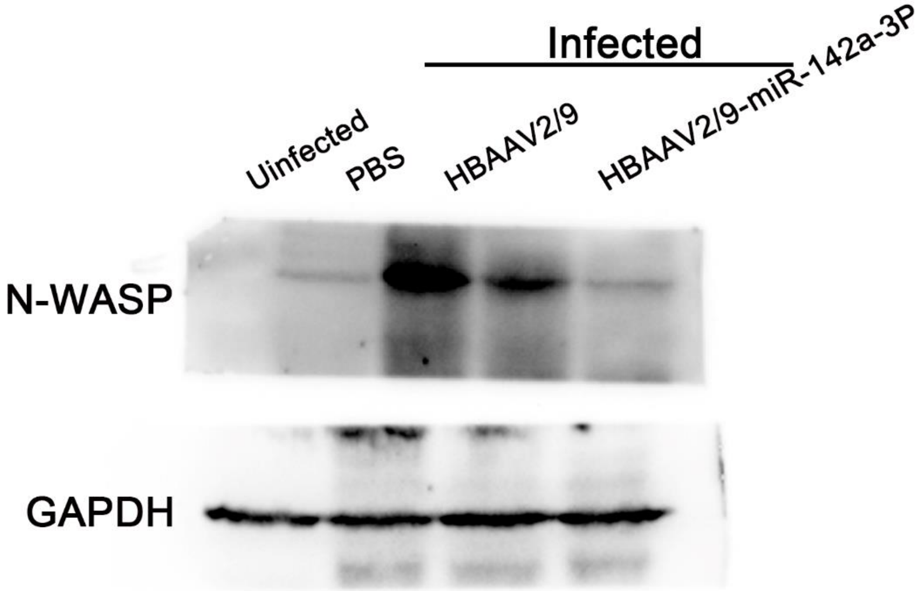

Figure 7B

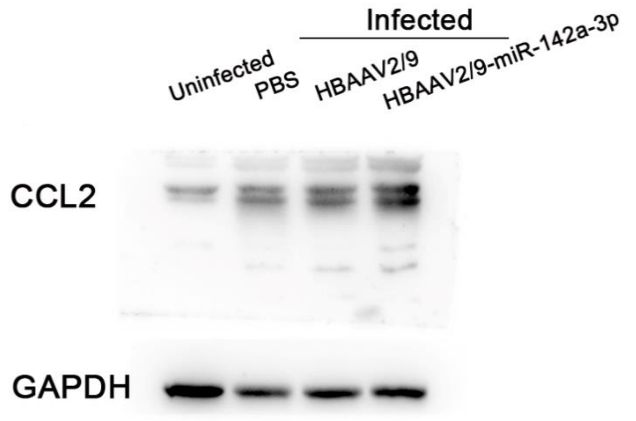

Figure 7D

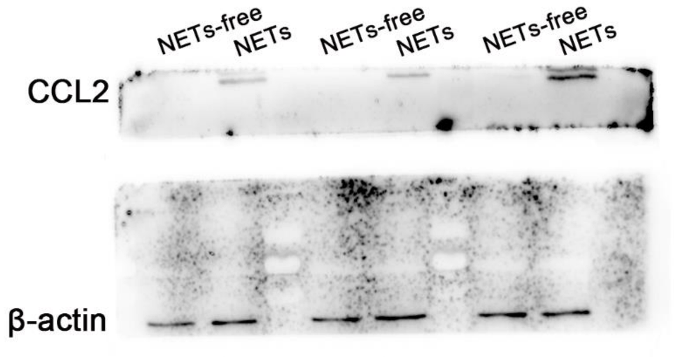

Figure 8E

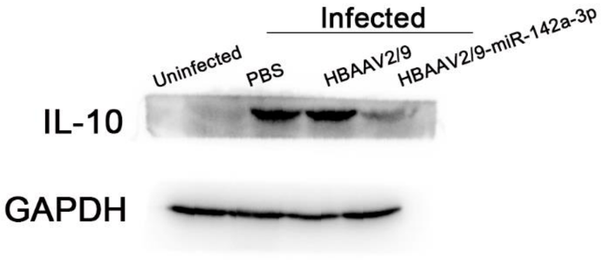

Figure 8K

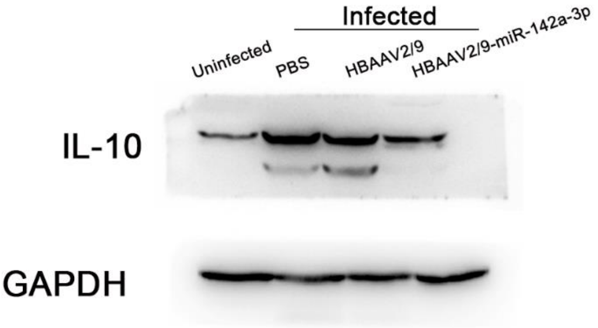

Figure 8L

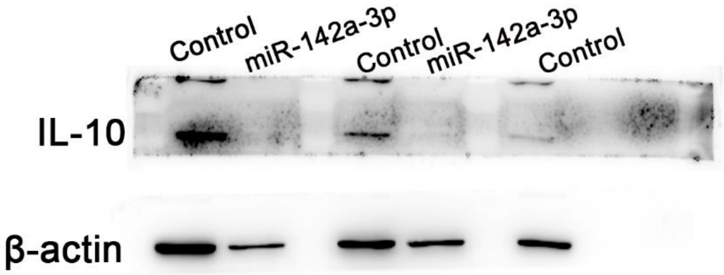

Figure 8N

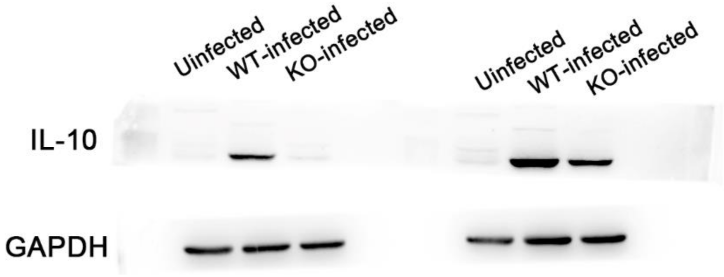

Supplement: Document S1. Figures S1–S7 and Tables S1 and S2 [file mmc1.pdf]
