# Supplementary material for: Host liver-derived extracellular vesicles deliver miR-142a-3p induces neutrophil extracellular traps via targeting WASL to block the development of Schistosoma japonicum
Source: Mol Ther. 2022 Mar 26;30(5):2092–107. doi: 10.1016/j.ymthe.2022.03.016 (PMC9092393; doi:10.1016/j.ymthe.2022.03.016)

Molecular Therapy
Original Article

# Host liver-derived extracellular vesicles deliver miR-142a-3p induces neutrophil extracellular traps via targeting WASL to block the development of *Schistosoma japonicum*

Lifu Wang,[2,3,8] Zifeng Zhu,[1,3,4,8] Yao Liao,[1,3,4] Lichao Zhang,[1,3,4] Zilong Yu,[5] Ruibing Yang,[6] Ji Wu,[7] Zhongdao Wu,[1,3,4] and Xi Sun[1,3,4]

[1]Department of Parasitology, Zhongshan School of Medicine, Sun Yat-sen University, Guangzhou 510080, China; [2]Guangzhou Key Laboratory for Clinical Rapid Diagnosis and Early Warning of Infectious Diseases, KingMed School of Laboratory Medicine, Guangzhou Medical University, Guangzhou, China; [3]Key Laboratory of Tropical Disease Control, Ministry of Education, Sun Yat-sen University, Guangzhou 510180, China; [4]Provincial Engineering Technology Research Center for Biological Vector Control, Guangzhou, China; [5]Guangdong Second Provincial General Hospital, Guangzhou 510320, China; [6]Guangzhou Kingmed Center for Clinical Laboratory, Guangzhou 510310, China; [7]Medical Department of Xizang Minzu University, Xianyang 712082, China

**Schistosomiasis is an important neglected tropical disease. Interactions between the host immune system and schistosomes are complex. Neutrophils contribute to clearance of large pathogens primarily by releasing neutrophil extracellular traps (NETs). However, the functional role of NETs in clearing schistosomes remains unclear. Herein, we report that extracellular vesicles (EVs) derived from the liver of *Schistosoma japonicum*-infected mice (IL-EVs) induce NET release by delivering miR-142a-3p to target WASL and block the development of *S. japonicum*. WASL knockout accelerated the formation of NETs that blocked further development of *S. japonicum*. miR-142a-3p and NETs upregulated the expression of CCL2, which recruits macrophages that block *S. japonicum* development. However, *S. japonicum* inhibited NET formation in wild-type mice by upregulating host interleukin-10 (IL-10) expression. In contrast, in WASL knockout mice, IL-10 expression was downregulated, and *S. japonicum*-mediated inhibition of NET formation was significantly reduced. IL-EV-mediated induction of NET formation is thus an anti-schistosome response that can be counteracted by *S. japonicum*. These findings suggest that IL-EV-mediated induction of NET formation plays a key role in schistosome infection and that WASL is a potential therapeutic target in schistosomiasis and other infectious diseases.**

## INTRODUCTION

Schistosomiasis is a neglected tropical disease that affects 240 million people globally and causes 250,000 deaths annually.[1,2] Female *Schistosoma japonicum* worms live in the mesenteric veins of host organisms and produce numerous eggs, many of which are transported to the liver. Highly immunogenic substances released by these eggs elicit host immune responses, including granulomatous inflammation and fibrotic reactions. Schistosomes can flourish in the host despite the development of a pronounced immune response. Unfortunately, no schistosomiasis vaccines are available, and praziquantel is the only drug available for treatment. Therefore, a better understanding of how the immune system responds to schistosomes may facilitate the development of effective therapeutic strategies.

In schistosomiasis, schistosomes evoke responses from many immune cells, including T helper cells, eosinophils, neutrophils, and macrophages, and induce chemokine release.[3] Neutrophils are the most abundant innate immune effector cells and play a pivotal role in eliminating invading pathogens.[4] Neutrophils are also believed to play an important role in *S. japonicum* granuloma formation.[5] The *S. japonicum* egg protein SjE16.7 recruits neutrophils and initiates inflammatory hepatic granuloma formation.[6] In schistosomiasis caused by *S. japonicum*, interferon-γ contributes to neutrophil recruitment and the granulomatous response.[7] Neutrophils are rapidly recruited to sites of infection and form the initial immunological barrier against pathogens via phagocytosis,[8] degranulation,[9] and the formation of neutrophil extracellular traps (NETs).[10] NETs are large, extracellular, web-like structures consisting of decondensed chromatin decorated with immunostimulatory and microbicidal molecules.[10–12] The "stickiness" of NETs enables efficient entrapment of pathogens to prevent further dissemination. Initial studies of NETs involved

Received 22 November 2021; accepted 24 March 2022;
https://doi.org/10.1016/j.ymthe.2022.03.016.

[8]These authors contributed equally

**Correspondence:** Xi Sun, Department of Parasitology, Zhongshan School of Medicine, Sun Yat-sen University, Guangzhou 510080, China.
**E-mail:** sunxi2@mail.sysu.edu.cn
**Correspondence:** Zhongdao Wu, Department of Parasitology, Zhongshan School of Medicine, Sun Yat-sen University, Guangzhou 510080, China.
**E-mail:** wuzhd@mail.sysu.edu.cn

 © 2022 The Authors.
This is an open access article under the CC BY license (http://creativecommons.org/licenses/by/4.0/).

bacteria,[11] fungi,[13] and viruses.[4] Neutrophil activation and NET release via autophagy represent one of the primary immunological defense mechanisms in severe acute respiratory syndrome coronavirus 2 (SARS-CoV-2) infection, and the resulting increased NETosis can lead to sustained inflammation.[14]

More recent studies have described NETs in protozoan infections and infections with some helminth parasites. In particular, one study showed that the protozoan pathogen *Toxoplasma gondii* elicits the formation of NETs, resulting in a subsequent decrease in *T. gondii* viability.[15] NETs drive inflammatory pathogenesis in malaria and promote *Plasmodium chabaudi* sequestration in the organs.[16] NETs may also contribute to containment of *Leishmania donovani* promastigotes, thereby facilitating their uptake by mononuclear phagocytes.[17] Human neutrophils co-cultured with *Strongyloides stercoralis* larvae induce the formation of NETs that ensnare the parasites and facilitate larval killing by immune cells.[18] Hookworms, however, secrete a deoxyribonuclease that degrades NETs.[19] Despite the above observations, knowledge regarding NET formation in response to parasites and the role of NETs in the resolution of parasitic diseases remains limited. The presence of infiltrating neutrophils in *S. japonicum*-induced granulomas was observed in a transcriptomic study of hepatic granulomas.[20] However, the role of neutrophils and subsequently formed NETs in schistosome infections remains poorly understood.

Extracellular vesicles (EVs) are defined as heterogeneous plasma membrane vesicles that contain large quantities of proteins, lipids, and nucleic acids, and function in the long-distance exchange of biochemical information.[21–24] Emerging evidence indicates that EVs play an important role in host-parasite communication as well. MicroRNAs (miRNAs) are a class of small, non-coding RNAs that negatively regulate gene expression at the post-transcriptional or translational levels via complementary binding to the 3′ untranslated region (UTR) of target mRNAs.[25–28] Recent studies have shown that EVs of *Schistosoma* species can carry miRNAs for specific communication with the host, and Zhu et al. showed that EVs derived from *S. japonicum* eggs contain small RNAs.[29] Meningher et al. showed that EVs derived from *Schistosoma mansoni* worms contain miRNAs that modulate host T helper cell differentiation.[30] We previously demonstrated that Sja-miR-71a in EVs derived from *Schistosoma* eggs inhibits liver fibrosis in *S. japonicum* infection.[31] However, little is known about the role of host-derived EVs in infections with *Schistosoma.*

In this study, we found that EVs derived from the liver of mice infected with *S. japonicum* (IL-EVs) induce the formation of NETs. The miRNA miR-142a-3p carried by IL-EVs plays a key role in IL-EV-mediated induction of NET formation via targeting of WASL. The resulting NETs block further development of *S. japonicum* in infected mice and attenuate the pathological progression of infection. However, *S. japonicum* is capable of inhibiting NET formation by upregulating the expression of interleukin-10 (IL-10).

## RESULTS

### IL-EVs induced NET formation

Released EVs were isolated from liver tissues of *S. japonicum*-infected and uninfected mice at 56 days (Table S1). EVs were confirmed by negative-staining transmission electron microscopy (TEM) as having a diameter ranging from 50 to 150 nm and a characteristic cup-shaped morphology (Figure 1A, arrows). Western blotting analysis confirmed expression of the EV surface markers CD9, CD63, and CD81 (Figure 1B). The size distribution profile of the EVs was examined by nanoparticle tracking analysis (NTA), which revealed peak sizes of 84 nm (normal liver-derived EVs [NL-EVs]) and 75 nm (IL-EVs) (Figure 1C).

A population of $CD11b^+Ly6G^+$ cells (neutrophils) surrounding the *S. japonicum* granuloma was observed (Figure 1D), and NL-EVs were internalized by neutrophils (Figure 1E). NETs, are released by neutrophils and neutralize and kill bacteria, fungi, viruses, and parasites.[10] Interestingly, IL-EVs (but not NL-EVs) induced significant NET formation (Figure 1F). In addition, IL-EVs (but not NL-EVs) promoted phorbol-12-myristate-13-acetate (PMA)-induced NET formation (Figure 1G). Collectively, these data suggest that, during infection with *S. japonicum*, host liver tissue secretes EVs to induce the formation of NETs.

### IL-EVs induced the formation of NETs via delivery of miR-142a-3p

miRNAs are major components carried by EVs and play prominent roles in regulating gene expression.[32] We therefore assessed the expression profiles of miRNAs in IL-EVs and NL-EVs (Figures S1A and S1B). Compared with NL-EVs, miR-142a-3p levels were significantly higher in IL-EVs (Table S2; Figure 2A). We then explored the potential role of miR-142a-3p in IL-EV-mediated induction of NET formation. Interestingly, miR-142a-3p induced significant NET formation (Figures 2B and 2C). In addition, miR-142a-3p promoted PMA-induced NET formation (Figure 2D). Furthermore, to assess the role of miR-142a-3p in IL-EV-induced NET formation, we treated cells with a miR-142a-3p inhibitor (chemically modified mature miR-142a-3p complementary single chain) to reduce miR-142a-3p levels in IL-EVs. IL-EV-mediated induction of NET formation decreased significantly after inhibition of miR-142a-3p (Figures 2E and 2F). Collectively, these results suggest that the induction of NET formation by IL-EVs is primarily mediated by the delivery of miR-142a-3p.

### miR-142a-3p induced NET formation and blocked further development of *S. japonicum*, thereby attenuating the pathological progression of *S. japonicum* infection

To determine whether miR-142a-3p delivery by IL-EVs is involved in the progression of schistosomiasis, HBAAV2/9-miR-142a-3p recombinant adeno-associated virus (rAAV) were constructed. Mice were infected with *S. japonicum* and exposed to HBAAV2/9-miR-142a-3p rAAV (Figure 3A). Liver sections reflected the highly hepatic tropism of HBAAV2/9-miR-142a-3p (Figure S2). As shown in Figure 3B, the number of $CD11b^+Ly6G^+$ neutrophils surrounding the

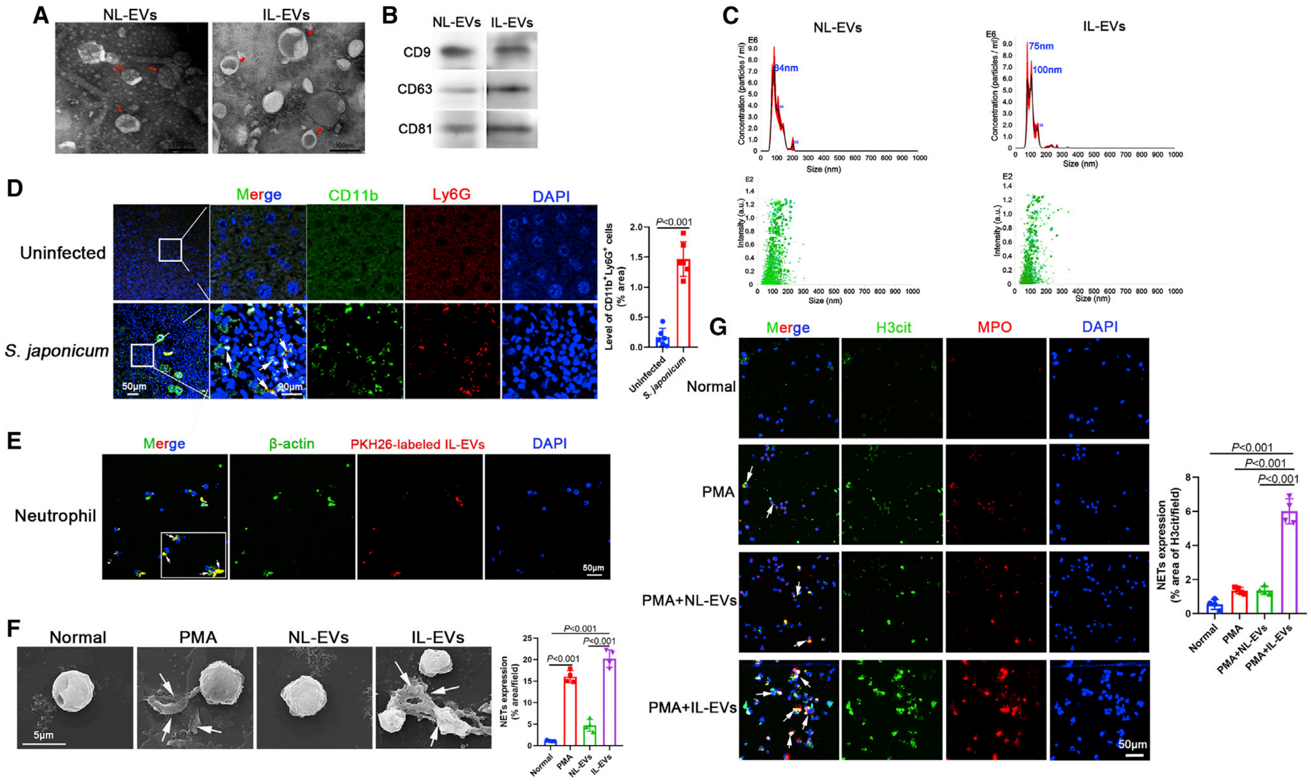


**Figure 1. EVs derived from the liver of *S. japonicum*-infected mice (IL-EVs) induced the formation of NETs**

(A) Normal liver-derived EVs (NL-EVs) and IL-EVs were purified and analyzed by negative-staining transmission electron microscopy. (B) Expression of EV surface markers (CD9, CD63, CD81) was analyzed by western blotting. (C) EV particles were investigated using nanoparticle tracking analysis (NTA). (D) Neutrophils in liver sections were examined for CD11b and Ly6G co-localization (n = 6 mice per group). (E) Neutrophils were incubated with PKH26-labeled NL-EVs, and EV internalization was examined using laser scanning confocal microscopy. (F) Neutrophils were treated with PMA (500 nM, 4 h), NL-EVs (10 μg/mL, 24 h), or IL-EVs (10 μg/mL, 24 h), and neutrophil extracellular traps (NETs) were observed using scanning electron microscopy (SEM). (G) Neutrophils were treated with PMA (500 nM, 4 h), NL-EVs (10 μg/mL, 24 h) + PMA (500 nM, 4 h), or IL-EVs (10 μg/mL, 24 h) + PMA (500 nM, 4 h), and NETs were detected based on H3cit and MPO co-localization. (D) Unpaired two-sample t test. (F and G) One-way ANOVA with Dunnett's multiple comparison test.

*S. japonicum* granuloma significantly increased after HBAAV2/9-miR-142a-3p treatment. We then analyzed NET formation in the liver of infected mice. As expected, NETs were observed in *S. japonicum* granulomas (Figure S3), and a significant increase in NET formation was observed in mice treated with HBAAV2/9-miR-142a-3p (Figure 3C). Neutrophils were isolated from infected mice using Percoll density gradient centrifugation and flow cytometry (Figure S4) and then cultured *in vitro* for 24 h. NETs formed by these neutrophils increased markedly after HBAAV2/9-miR-142a-3p transfection (Figure 3D), and HBAAV2/9-miR-142a-3p promoted PMA-induced NET formation (Figure 3E).

To obtain a better understanding of the effect of miR-142a-3p on IL-EV-induced NET formation in the progression of schistosomiasis, characteristics of the worms were examined. As shown in Figure 4A, development of both male and female worms was blocked by HBAAV2/9-miR-142a-3p treatment. Furthermore, worms collected from mice treated with HBAAV2/9-miR-142a-3p were significantly shorter in length than those obtained from mice treated with either PBS or HBAAV2/9 (Figure 4A). Furthermore, following exposure to HBAAV2/9-miR-142a-3p, a significant decrease in egg deposition in the liver was observed (Figure 4B). Hepatosplenomegaly was markedly attenuated after HBAAV2/9-miR-142a-3p treatment, and the liver and spleen indices declined significantly after HBAAV2/9-miR-142a-3p treatment (Figures 4C and 4D). In addition, thymus atrophy caused by *S. japonicum* infection diminished following HBAAV2/9-miR-142a-3p treatment (Figure 4E). Hematoxylin and eosin (H&E) staining of liver sections revealed that the percent egg granuloma area and area of a single granuloma declined significantly in the HBAAV2/9-miR-142a-3p-treated groups compared with the PBS- and HBAAV2/9-treated groups (Figures 4F and 4G). Masson's trichrome staining of liver sections showed significantly reduced collagen deposition in HBAAV2/9-miR-142a-3p-treated mice (Figure S5A). Similarly, significantly reduced expression of α-SMA and collagen I was observed in HBAAV2/9-Sja-miR-71a-treated mice (Figures S5B and S5C). The high expression of the inflammatory cytokines IL-1β, IL-23A, and IL-33, and tumor necrosis factor alpha (TNF-α) associated with

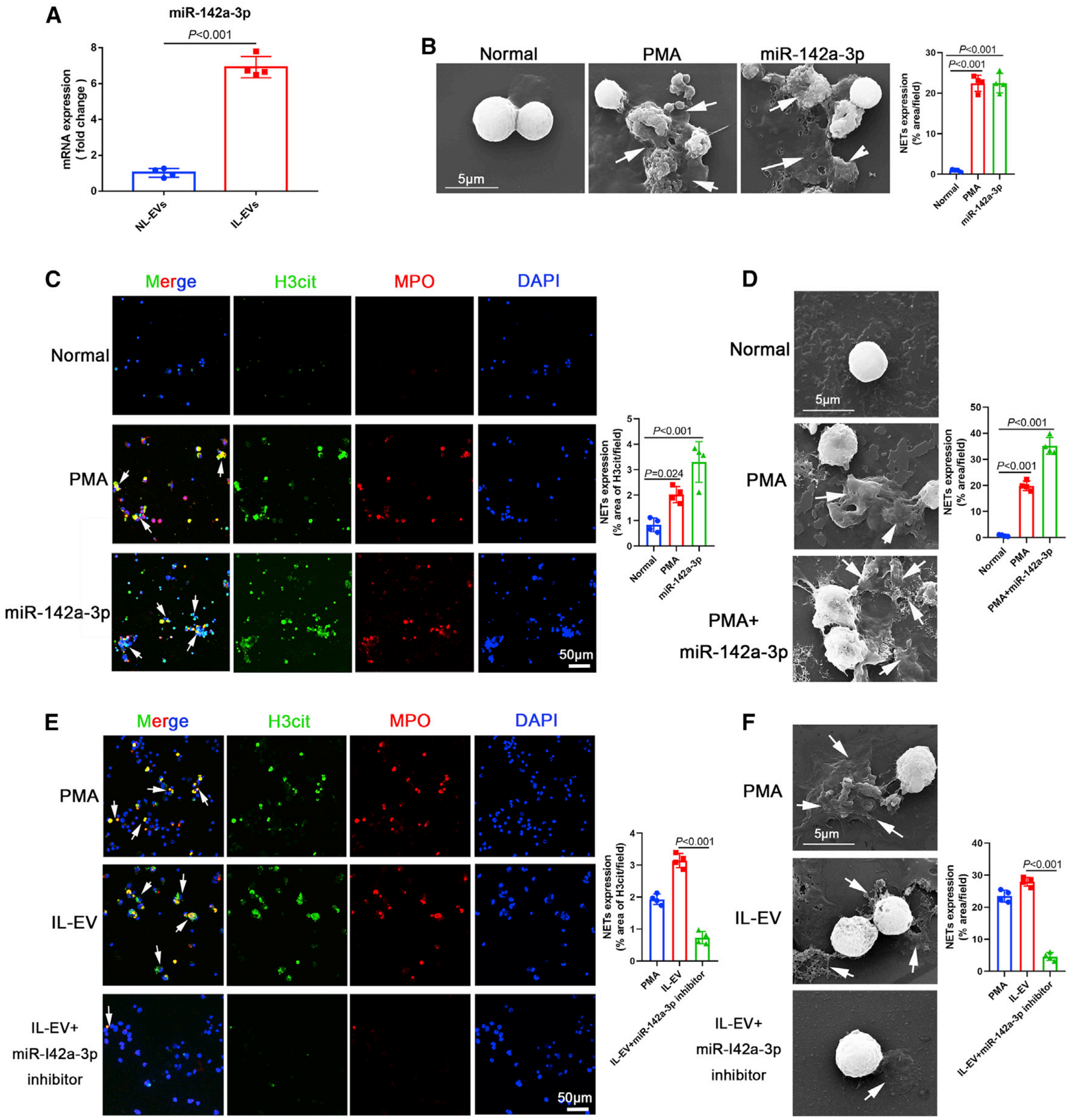


**Figure 2. IL-EVs induced the formation of NETs via the delivery of miR-142a-3p**

(A) miR-142a-3p levels in EVs were determined using quantitative real-time polymerase chain reaction (qRT-PCR). (B) Neutrophils were treated with PMA (500 nM, 4 h) and miR-142a-3p (50 nM, 24 h), and NETs were observed using SEM. (C) Neutrophils were treated with PMA (500 nM, 4 h) and miR-142a-3p (50 nM, 24 h), and NETs were detected based on H3cit and MPO co-localization. (D) Neutrophils were treated with PMA (500 nM, 4 h) and miR-142a-3p (50 nM, 24 h) + PMA (500 nM, 4 h), and NETs were observed using SEM. (E and F) Neutrophils were treated with PMA (500 nM, 4 h), IL-EVs (10 μg/mL, 24 h), and IL-EVs (10 μg/mL, 24 h) + miR-142a-3p inhibitor (100 nM, 24 h), and NETs were detected by immunohistochemistry and observed using SEM. (A) Unpaired two-sample t test. (C–F) One-way ANOVA with Dunnett's multiple comparison test.

*S. japonicum* infection decreased significantly after HBAAV2/9-miR-142a-3p treatment (Figures S6A–S6D).

Taken together, these results suggest that IL-EVs induce NET formation by delivering miR-142a-3p, which blocks further development of *S. japonicum* and attenuates the pathological progression of schistosomiasis.

#### miR-142a-3p induced NET formation via direct targeting of WASL

To further evaluate the molecular mechanisms involved in miR-142a-3p-mediated induction of NET formation, we identified genes directly targeted and thereby controlled by miR-142a-3p. TargetScan was used to predict potential miR-142a-3p targets. We found that expression of WASL in the liver of *S. japonicum*-infected mice declined after

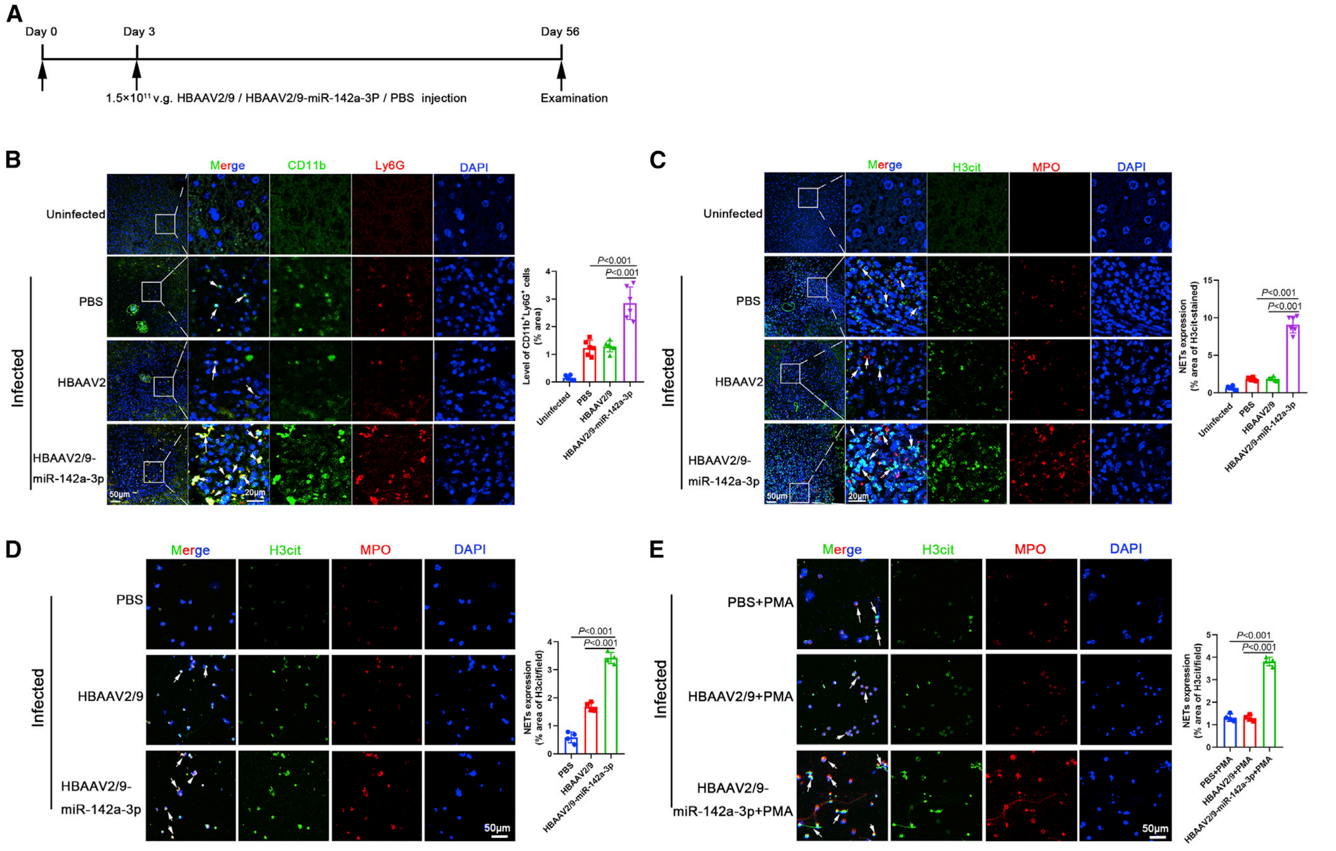


**Figure 3. miR-142a-3p in IL-EVs induced NET formation**

(A) Time schedule for parasite infection and intravenous injection of rAAV vectors or PBS and sample examination. (B) Neutrophils in liver sections were detected based on CD11b and Ly6G co-localization (n = 6 mice per group). (C) NETs in liver sections were detected based on H3cit and MPO co-localization (n = 6 mice per group). (D) Neutrophils were isolated from infected mice using Percoll density gradient centrifugation and flow cytometry, and cultured *in vitro* for 24 h. NETs were then detected based on H3cit and MPO co-localization. (E) Neutrophils were isolated from infected mice and treated with PMA (500 nM) for 4 h and then detected based on H3cit and MPO co-localization. Differences were analyzed using one-way ANOVA with Dunnett's multiple comparison test.

HBAAV2/9-miR-142a-3p treatment (Figure 5A). Furthermore, as shown in Figure 5B, miR-142a-3p targeted four binding sites in the 3′ UTR of WASL. To confirm WASL as a direct target of miR-142a-3p, we generated a luciferase reporter plasmid containing the 3′ UTR of WASL and flanking putative miR-142a-3p-binding sites and a plasmid mutated miRNA binding sites. Dual-luciferase reporter assay results revealed that, compared with normal control mimic-transfected cells, cells transfected with miR-142a-3p mimic exhibited significantly reduced luciferase activity of the WASL construct (Figure 5B). In contrast, the miR-142a-3p mimic had no effect on the luciferase activity of the WASL construct with mutated miR-142a-3p-binding sites (Figure 5B).

We analyzed the expression of neural Wiskott-Aldrich syndrome protein (N-WASP, translated from WASL) in mouse liver. As expected, N-WASP expression was markedly downregulated in the liver of mice treated with HBAAV2/9-miR-142a-3p compared with control mice (Figures 5C and 5D). N-WASP expression was also evaluated in neutrophils of infected mice. As shown in Figure 5E, neutrophil N-WASP expression was downregulated following HBAAV2/9-miR-142a-3p treatment. In addition, to evaluate the role of N-WASP in the miR-142a-3p-induced NET formation, N-WASP expression was inhibited with Wiskostatin, which resulted a significant increase in PMA-induced NET formation (Figures 5F and 5G). Taken together, these findings indicate that miR-142a-3p induces NET formation by directly targeting WASL.

## WASL deletion accelerated NET formation and blocked further development of *S. japonicum*

The key role of WASL in NET formation was examined using neutrophils isolated from wild-type (WT) and $WASL^{-/-}$ mice. Interestingly, WASL deletion significantly accelerated NET formation compared with WT mice (Figure 6A). In addition, PMA-induced NET formation was greater in $WASL^{-/-}$ mice than WT mice (Figure 6B). Next, we sought to gain a better understanding of miR-142a-3p-induced NET formation by directly targeting WASL. Liver sections of WASL knockout (WASL-KO) mice infected with *S. japonicum* exhibited a significant increase in $CD11b^{+}Ly6G^{+}$ neutrophils surrounding

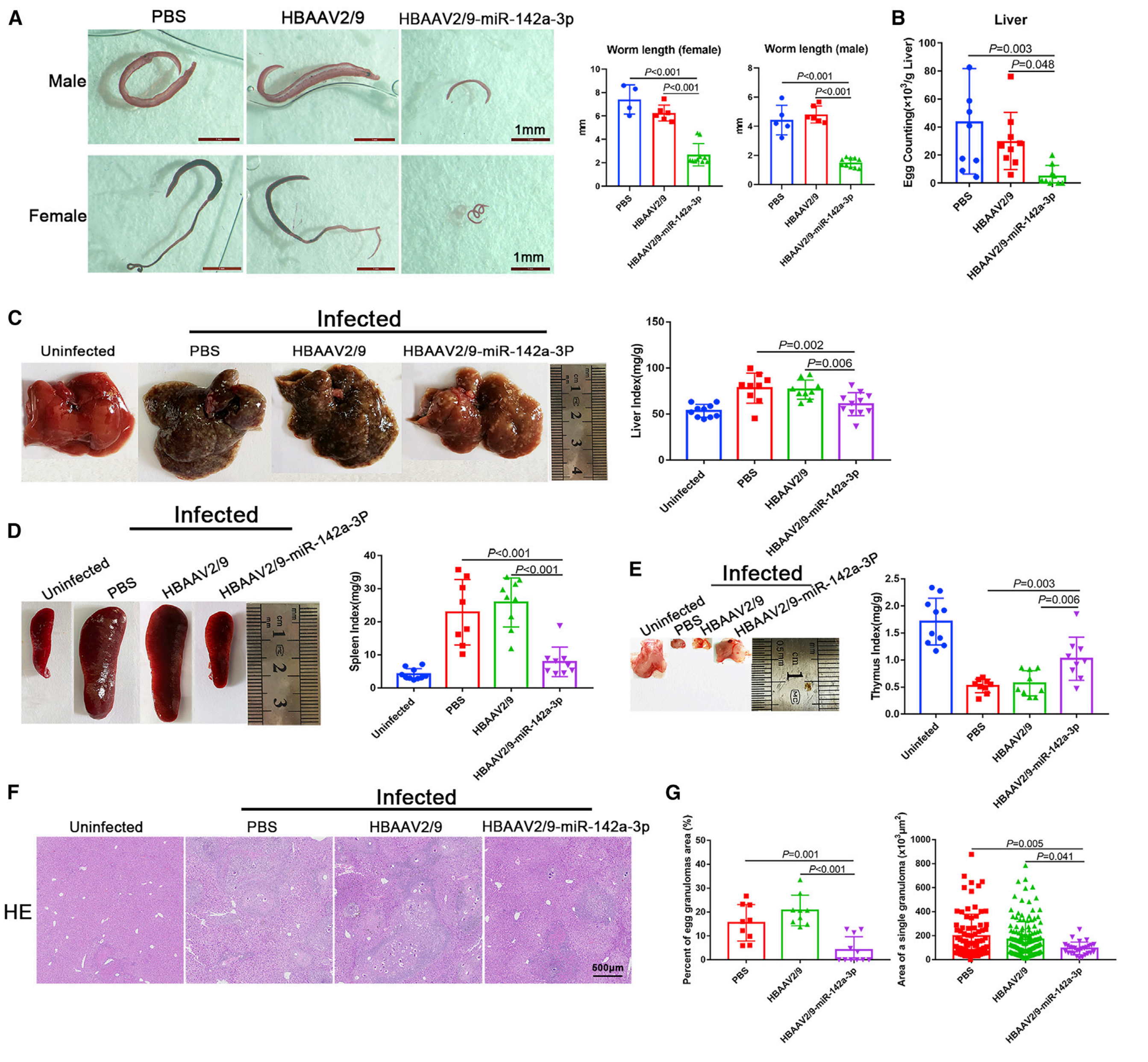


**Figure 4. miR-142a-3p induced NET formation to block further development of *S. japonicum* and attenuated the pathological progression of schistosomiasis**

(A) Worms were stained with hydrochloric carmine red to assess development and length. (B) Liver egg burden was determined after digesting liver tissue in 4% KOH (n = 8–9 mice per group). (C–E) Macroscopic appearance of the liver, spleen, and thymus and related indices (n = 8–11 mice per group). (F) Hematoxylin and eosin (H&E) staining of liver sections. (G) Percent granulomatous area and area of a single granuloma were measured from H&E sections using Image Pro Plus 6.0 software (n = 9–11 mice per group). Differences were analyzed using one-way ANOVA with Dunnett's multiple comparison test.

*S. japonicum* granulomas (Figure 6C). We then analyzed NET formation in the liver of infected WASL-KO mice. As expected, compared with WT mice, NET formation was significantly increased in the liver of WASL-KO mice (Figure 6D). Neutrophils were isolated from infected mice and cultured *in vitro* for 24 h, and we observed a marked increase in NETs after WASL deletion (Figure 6E). WASL deletion promoted PMA-induced NET formation (Figure 6F).

To further confirm that WASL deletion affects the progression of schistosomiasis by promoting NET formation, characteristics of the worms were examined. As shown in Figure 6G, after 4 weeks of

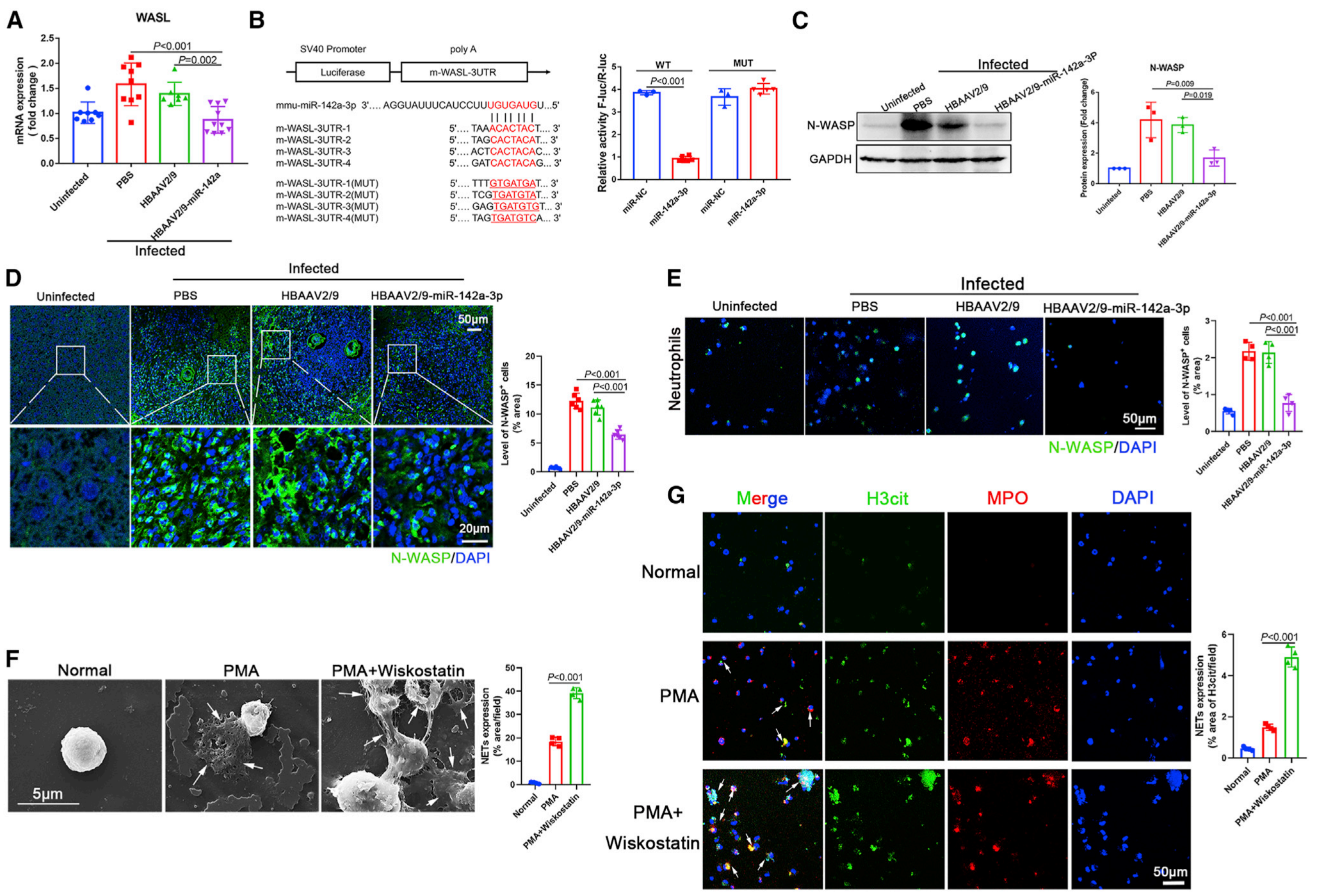

**Figure 5. miR-142a-3p induced NET formation via direct targeting of WASL**

(A) WASL mRNA expression was analyzed by qRT-PCR (n = 7–10 mice per group). (B) Wild-type and mutated m-WASL-3′ untranslated regions (UTRs) were cloned into psi-CHECK-2, and four predicted binding sites of miR-142a-3p were identified in the 3′ UTR of the WASL gene; dual-luciferase reporter assay performed on HEK293T cells transfected with WASL UTR reporter plasmid together with miR-142a-3p mimic or control mimic. (C and D) Expression of N-WASP (translated from WASL) in mice livers was analyzed by western blotting and immunohistochemistry (n = 6 mice per group). (E) Neutrophils of infected mice were isolated, and the expression of N-WASP was analyzed by immunohistochemistry. (F and G) Neutrophils were treated with PMA (500 nM, 4 h) or Wiskostatin (N-WASP inhibitor, 20 μM, 24 h) + PMA (500 nM, 4 h), and NET formation was evaluated by SEM and immunohistochemistry. (A, C, and D–G) One-way ANOVA with Dunnett's multiple comparison test. (B) Unpaired two-sample t test.

*S. japonicum* infection, the body weight of WT mice decreased dramatically, whereas the body weight of WASL-KO mice did not change significantly compared with uninfected mice. We found that worm development was blocked by WASL deletion. Worms collected from WASL-KO mice were significantly shorter than those obtained from WT mice (Figure 6H), and a significant decrease in egg deposition in the liver was observed in WASL-KO mice (Figure 6I). Furthermore, hepatomegaly was markedly attenuated in WASL-KO mice, and the liver index was significantly reduced (Figure 6J). H&E staining of liver sections revealed that the percent egg granuloma area and the area of a single granuloma were significantly reduced in WASL-KO mice compared with WT mice (Figure 6K). Masson's trichrome staining of liver sections showed significantly reduced collagen deposition in WASL-KO mice (Figure S7A), along with significantly reduced expression of α-SMA and collagen I (Figure S7B). Taken together, these data suggest that WASL deletion accelerates the formation of NETs, which then function to block further development of *S. japonicum.*

### miR-142a-3p and NETs upregulated CCL2 to recruit macrophages to block further *S. japonicum* development

Macrophages contribute significantly to the immune response during helminth infection. Alternatively activated macrophages are induced early in the anti-helminth immune response.[33] We observed numerous macrophages adhering to the surface of *S. japonicum* worms after HBAAV2/9-miR-142a-3p treatment (Figure 7A, arrows). Furthermore, expression of CCL2, a potent chemotactic factor for monocytes, was markedly upregulated after HBAAV2/9-miR-142a-3p treatment (Figure 7B). *In vitro* experiments also revealed that miR-142a-3p treatment significantly upregulated the expression of CCL2 in macrophages (Figure 7C). In addition, NETs markedly upregulated the expression of CCL2 in macrophages (Figure 7D).

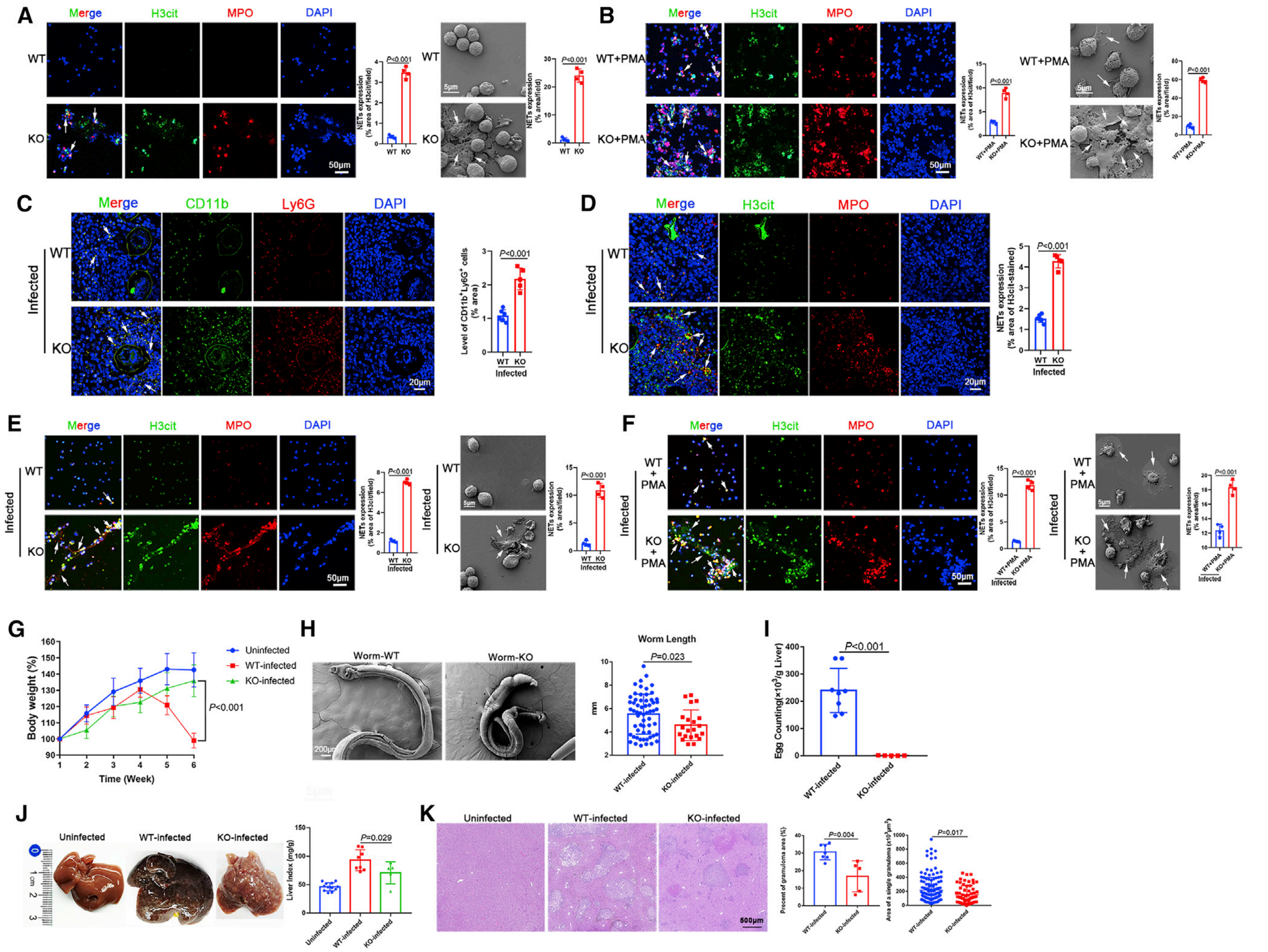

**Figure 6. WASL deletion accelerated NET formation and blocked further development of *S. japonicum***

(A) Neutrophils were cultured *in vitro* for 24 h, and NETs were observed by immunohistochemistry and SEM. (B) Neutrophils were treated with PMA (500 nM, 4 h), and NETs were then observed by immunohistochemistry and SEM. (C) Observation of neutrophils in liver sections (n = 6 mice per group). (D) NETs in liver sections were detected based on H3cit and MPO co-localization (n = 6 mice per group). (E) Neutrophils were isolated from WT and WASL knockout (WASL-KO) mice infected with *S. japonicum* (6 weeks) and cultured *in vitro* for 24 h. NETs were then observed by immunohistochemistry and SEM. (F) Neutrophils were isolated from WT and WASL-KO mice infected with *S. japonicum* (6 weeks) and treated with PMA (500 nM) for 4 h, after which NETs were observed by immunohistochemistry and SEM. (G) Body weight of mice was recorded weekly (n = 5–12 mice per group). (H) Observation and measurement of *S. japonicum* worm length using SEM. (I) Determination of liver egg burden (n = 5–8 mice per group). (J) Macroscopic appearance of the liver and liver index (n = 5–12 mice per group). (K) The granulomatous area was measured from H&E sections (n = 5–7 mice per group). (A–F, H, I, and K) Unpaired two-sample t test. (G and J) One-way ANOVA with Dunnett's multiple comparison test.

Collectively, these data suggest that miR-142a-3p and NETs upregulate CCL2 expression to recruit macrophages to block further development of *S. japonicum.*

### *S. japonicum* inhibited NET formation by upregulating host IL-10 expression, whereas miR-142a-3p downregulated the expression of IL-10 by targeting WASL

Although neutrophils are upregulated during *S. japonicum* infection, the worms are not killed. We therefore hypothesized that *S. japonicum* inhibits NET formation. Thus, we co-cultured *S. japonicum* with PMA-stimulated neutrophils, and the results confirmed our hypothesis that *S. japonicum* inhibits PMA-induced NET formation (Figure 8A). Furthermore, as shown in Figures 8B and 8C, both *S. japonicum* soluble worm antigenic preparation (SWAP) and *S. japonicum* soluble eggs antigen (SEA) inhibited PMA-induced NET formation.

*S. japonicum* also significantly upregulated the expression of IL-10 in the host (Figures 8D and 8E). In *in vitro* experiments, SEA significantly upregulated the expression of IL-10 in macrophages (Figure 8F), and PMA-induced NET formation was subsequently inhibited by IL-10 (Figures 8G and 8H). Interestingly, IL-EVs

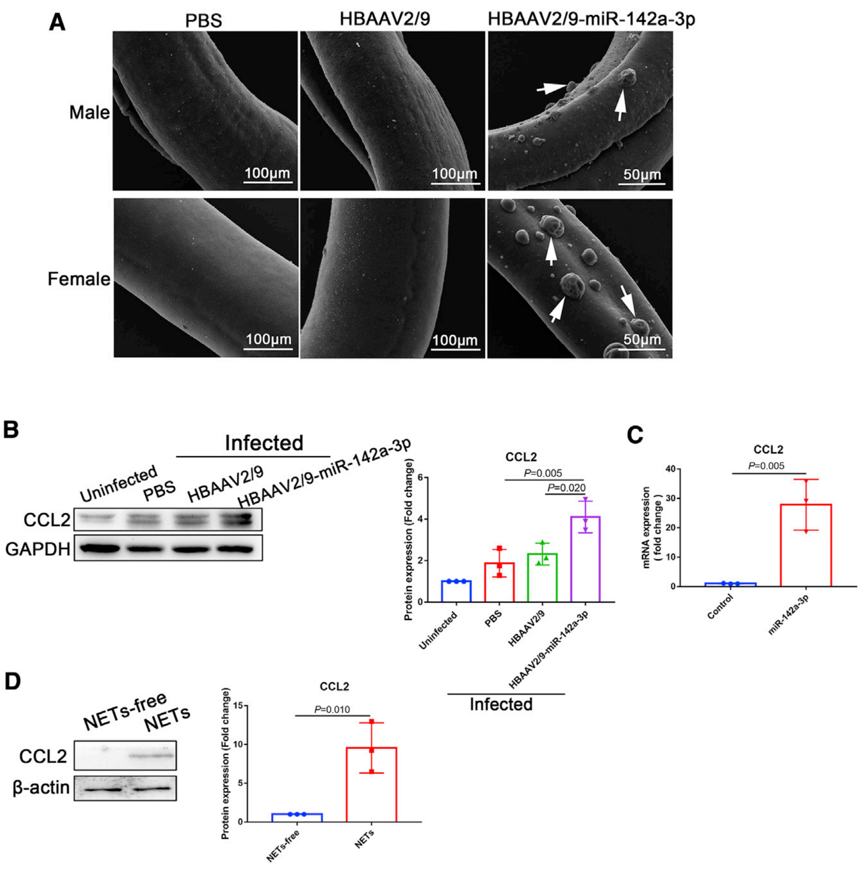


**Figure 7. miR-142a-3p and NETs upregulated CCL2 expression to recruit macrophages to block further development of *S. japonicum***

(A) *S. japonicum* worms were collected from infected mice and analyzed using SEM. (B) Expression of CCL2 in the liver was analyzed by western blotting. (C) Macrophages were treated with miR-142a-3p (50 nM, 24 h), and CCL2 expression was analyzed by qRT-PCR. (D) Macrophages were treated with NETs (10 μg/mL, 24 h), and CCL2 expression was analyzed by western blotting. (B) One-way ANOVA with Dunnett's multiple comparison test. (C and D) Unpaired two-sample t test.

downregulated the expression of IL-10 in macrophages (Figure 8I). Furthermore, *in vivo* experiments showed that IL-10 expression was upregulated in the liver by *S. japonicum* but downregulated after HBAAV2/9-miR-142a-3p treatment (Figures 8J and 8K). In addition, miR-142a-3p significantly downregulated the expression of IL-10 in macrophages *in vitro* (Figure 8L). To investigate whether miR-142a-3p downregulates the expression of IL-10 by targeting WASL, we determined the expression level of IL-10 in WASL-KO mice infected with *S. japonicum*. IL-10 expression was significantly reduced in WASL-KO mice compared with WT mice (Figures 8M and 8N). Similarly, compared with the WT mice shown in Figures 8B and 8C, the inhibitory effects of SWAP and SEA on PMA-induced NET formation in WASL-KO mice were significantly attenuated (Figures 8O and 8P).

Collectively, these data suggest that *S. japonicum* inhibits NET formation by upregulating host IL-10 expression to escape the host immune response, whereas miR-142a-3p contained in IL-EVs downregulates the expression of IL-10 by targeting WASL.

## DISCUSSION

In this study, we demonstrate that host EVs and EVs carrying miR-142a-3p contribute to block further development of *S. japonicum* in infected mice and attenuate the pathological progression of *S. japonicum* infection by inducing NET formation. Our data showed that IL-EVs contain high levels of miR-142a-3p, which induces NET formation by directly targeting WASL. WASL KO accelerates the formation of NETs that block further development of *S. japonicum*. In addition, miR-142a-3p and NETs upregulate CCL2 expression, leading to recruitment of macrophages that also block development of *S. japonicum*. We also demonstrated that *S. japonicum* inhibits NET formation by upregulating host IL-10 expression, whereas miR-142a-3p in IL-EVs downregulates the expression of IL-10 by targeting WASL (Figure 9).

The interactions between the host immune system and schistosomes is complex. The host immune system likely plays an important role in suppressing the enormous reproductive potential of schistosomes.[34] Immune responses against schistosomes are important for the development of resistance to reinfection.[35] Granulomas are a collection of inflammatory cells that include lymphocytes, eosinophils, macrophages, and neutrophils.[35] Granulomas induced by *S. japonicum* are primarily composed of neutrophils, whereas the core of hepatic granulomas induced by *S. mansoni* is composed primarily of eosinophils.[3] Hsü et al. first reported that *S. japonicum* egg lesions induce a stronger neutrophilic chemotaxis response than *S. mansoni* egg lesions.[36] Neutrophils are recruited to the areas surrounding *S. japonicum* eggs as early as 8 days post-deposition.[37] In this study, we identified a group of $CD11b^{+}Ly6G^{+}$ cells (neutrophils) surrounding granulomas of *S. japonicum*. However, the role of neutrophils in schistosome infection is not fully understood. NETs, which are released by neutrophils, are highly "sticky" and can capture bacteria,[11,38] fungi,[13] and viruses[4] to prevent their dissemination. Studies of patients with coronavirus disease 2019 (COVID-19) have shown that elevated levels of NET-specific markers (e.g., free DNA, MPO, and H3cit) are strongly associated with total neutrophil count.[39] SARS-CoV-2-triggered NETs mediate the pathology of COVID-19.[40] NETs contribute to immunothrombosis in COVID-19 acute respiratory distress syndrome.[41] Targeting NETs may therefore reduce the clinical severity of COVID-19.[42]

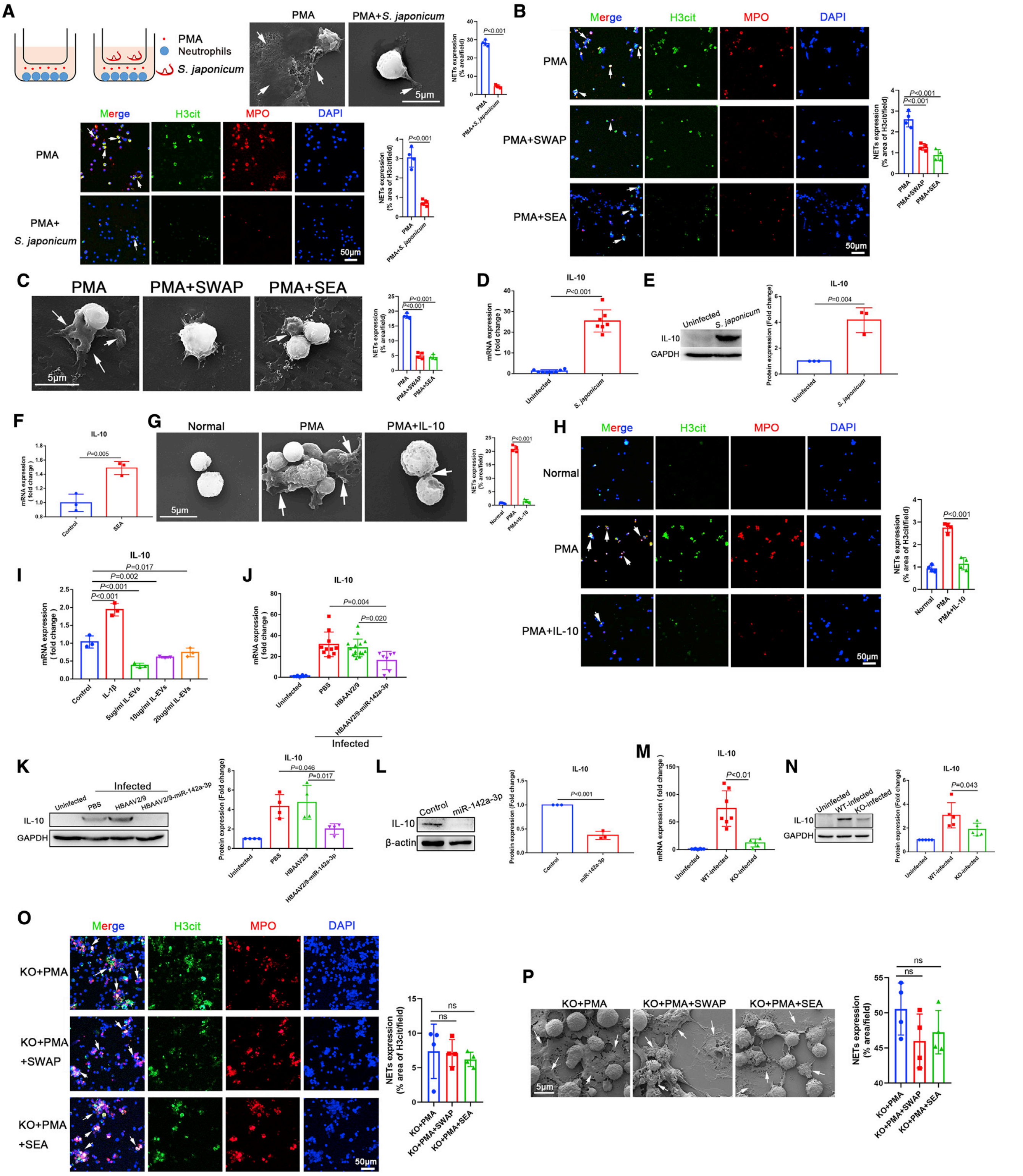


**Figure 8. *S. japonicum* inhibited NET formation by upregulating host IL-10 expression, whereas miR-142a-3p in IL-EVs downregulated IL-10 expression**

(A) *S. japonicum* worms were co-cultured with PMA-simulated (500 nM, 4 h) neutrophils ($5 \times 10^5$ cells) using Transwells, and NETs were observed by SEM and immunohistochemistry. (B and C) Neutrophils were treated with PMA (500 nM, 4 h), SWAP (10 μg/mL, 24 h) + PMA (500 nM, 4 h), or SEA (10 μg/mL, 24 h) + PMA (500 nM, 4 h), and

*(legend continued on next page)*

A number of recent studies have reported the presence of NETs in infections involving protozoans and a few helminth parasites, including *T. gondii*,[15] *L. donovani*,[17] *Plasmodium*,[43] *S. stercoralis*,[18] and *Haemonchus contortus*.[44] Chuah et al. reported observing NET-like structures surrounding *S. japonicum* eggs.[20] Nevertheless, the role of NETs in schistosome infection remains largely unknown.

Recent studies have also shown that EVs secreted from *S. japonicum* play important roles in host-parasite communication.[29–31] However, little is known about the role of host-derived EVs in schistosome infection. We hypothesized that host-derived EVs also play important roles in host-schistosome communication. Interestingly, we isolated EVs from the liver of *S. japonicum*-infected mice (i.e., IL-EVs) by ultracentrifugation and found that they induce NET formation. Among the different active components harbored by EVs, miRNAs are regarded as essential to EVs function.[45,46] To investigate the role of the key components in IL-EVs that induce NET formation, we sequenced and analyzed IL-EV miRNAs. Compared with NL-EVs, we found that the expression of miR-142a-3p was significantly increased in IL-EVs.

miR-142-3p was originally described as a hematopoietic-specific miRNA.[47] Studies have indicated that miR-142-3p is required for hematopoietic development. In *Xenopus*, knockdown of miR-142-3p impairs the specification of definitive hemangioblasts.[48] Furthermore, miR-142-3p plays a prominent role in $CD25^{+}CD4^{+}$ T cell proliferation by targeting the expression of glycoprotein A repetitions.[49] In zebrafish and mice, miR-142-3p is specifically expressed in hematopoietic stem cells, and knockdown of miR-142a-3p in zebrafish leads to a reduced population of hematopoietic stem cells.[50] In zebrafish, miR-142-3p also acts as an essential modulator of neutrophil development.[51] However, no studies have demonstrated an association between miR-142-3p and NET formation. Interestingly, we found that miR-142a-3p plays a key role in IL-EV-induced NET formation, whereas IL-EV-induced NET formation is significantly decreased following inhibition of miR-142a-3p.

Several studies have shown that N-WASP (encoded by the *Wasl* gene) plays an important role in B cell. The combined activity of N-WASP and WASP is required for peripheral B cell development and function.[52] For example, N-WASP is required for B cell-mediated autoimmunity in Wiskott-Aldrich syndrome.[53] Deletion of N-WASP and WASP in B cells results in the production of IgM autoantibodies.[54] However, the role of N-WASP in the development and function of neutrophils remains unknown. We demonstrated that miR-142a-3p induces NET formation by directly targeting WASL, which blocks further development of *S. japonicum* and attenuates the pathological progression of *S. japonicum* infection. Consistent with these observations, we found that WASL deletion accelerates NET formation and blocks the development of *S. japonicum*.

Macrophages contribute significantly to the immune response during helminth infection. Indeed, alternatively activated macrophages are induced early in the anti-helminth response.[33] We observed numerous macrophages adhering to the surface of worms after HBAAV2/9-miR-142a-3p treatment. Expression of CCL2, a potent chemotactic factor for monocytes,[55] was markedly upregulated after miR-142a-3p treatment, suggesting that miR-142a-3p and NETs upregulate CCL2 expression to recruit macrophages to block further development of *S. japonicum.*

Hepatic fibrosis is one of the primary pathological features of schistosomiasis. Female *S. japonicum* worms produce eggs that are transported to the liver, and these eggs elicit host immune responses and fibrotic reactions.[56] The function of neutrophils varies in schistosome-induced fibrosis depending on when they are recruited and their location in the granuloma.[3] In this study, we found that IL-EVs deliver miR-142a-3p to induce NET formation by targeting WASL, thereby blocking further development of *S. japonicum*. This causes egg deposition in the liver to decrease significantly, thereby alleviating fibrosis. In schistosome infection, macrophages in the periphery of granulomas exhibit matrix-degrading properties, and they may promote the recruitment of neutrophils.[3] We found that miR-142a-3p and NETs upregulate CCL2 expression to recruit macrophages and thereby block further development of *S. japonicum* and alleviate host fibrosis.

The host immune system works toward killing schistosomes; accordingly, schistosomes have developed immunoregulatory mechanisms to counteract the host immune response and enable the schistosomes to persist and complete their life cycle.[57] Interestingly, we observed that *S. japonicum* inhibited NET formation. Furthermore, both SWAP and SEA also inhibited NET formation, which may explain why neutrophils are upregulated during schistosome infection, but the worms are not killed. However, the development of *S. japonicum* was blocked after overexpression of miR-142a-3p, resulting in the recruitment of large numbers of neutrophils and inducing NET formation. These findings suggest that miR-142a-3p may be an important target for preventing schistosome infection.

NETs were observed by SEM and immunohistochemistry. (D and E) Expression of IL-10 in liver tissues was analyzed by qRT-PCR (n = 5–8 mice per group) and western blotting. (F) Macrophages were treated with SEA (10 μg/mL, 24 h), and IL-10 expression was analyzed by qRT-PCR. (G and H) Neutrophils were treated with PMA (500 nM, 4 h) or IL-10 (40 ng/mL, 24 h) + PMA (500 nM, 4 h), and NETs were observed by SEM and immunohistochemistry. (I) Macrophages were treated with IL-EVs, with IL-1β (20 ng/mL, 24 h) used as a positive control, and expression of IL-10 was analyzed by qRT-PCR. (J and K) Expression of IL-10 in the liver was analyzed by qRT-PCR (n = 7–16 mice per group) and western blotting. (L) Macrophages were treated with miR-142a-3p (50 nM, 24 h), and the expression of IL-10 was analyzed by western blotting. (M and N) Expression of IL-10 in mouse liver was analyzed by qRT-PCR (n = 5–8 mice per group) and western blotting. (O and P) Neutrophils from WASL-KO mice were treated with PMA (500 nM, 4 h), SWAP (10 μg/mL, 24 h) + PMA (500 nM, 4 h), or SEA (10 μg/mL, 24 h) + PMA (500 nM, 4 h), and NETs were observed by immunohistochemistry and SEM. (A, D, E, F, and L) Unpaired two-sample t test. (B, C, G–K, and M–P) One-way ANOVA with Dunnett's multiple comparison test.

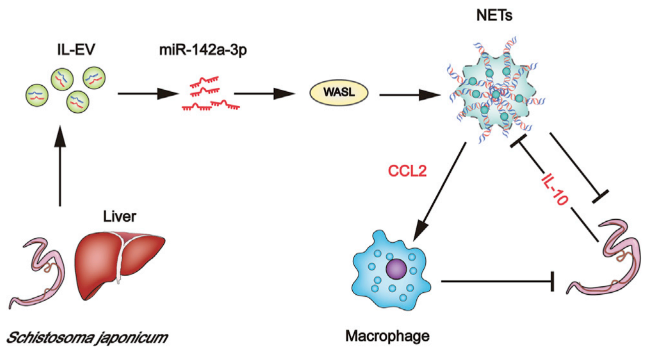


**Figure 9. Host liver-derived EVs deliver miR-142a-3p, which induces NET formation by targeting WASL to block further development of *S. japonicum***
NETs additionally upregulate CCL2 expression to recruit macrophages that also block development of *S. japonicum*. Nevertheless, *S. japonicum* can inhibit NET formation in wild-type mice by upregulating host IL-10 expression.

Helminths produce numerous specialized molecules that alter the host microenvironment and affect specific types of immune cells. Helminth infection also induces elevated expression of immunosuppressive cytokines, such as IL-10.[58] A previous study showed that expression of IL-4 induced primarily by schistosome eggs in turn induces IL-10 production by B cells.[57] Our previous study found that Sj16, a 16-kDa secreted protein of *S. japonicum*, significantly stimulates IL-10 production in dendritic cells.[59] The upregulation of IL-10 promotes Treg cell development, shifting the host immune response to a downregulated phenotype.[57] We found that *S. japonicum* significantly upregulates the expression of IL-10 in the host, which subsequently inhibits NET formation. The level of IL-10 was downregulated after treatment with miR-142a-3p. IL-10 expression was significantly decreased in WASL-KO mice, and the inhibitory effects of SWAP and SEA on PMA-induced NET formation were significantly diminished in WASL-KO mice. These data suggest that *S. japonicum* inhibits NET formation by upregulating host IL-10 expression to escape the host immune response, whereas miR-142a-3p downregulates the expression of IL-10 by directly targeting WASL.

### Conclusion
In conclusion, we demonstrated that IL-EVs deliver miR-142a-3p to induce the formation of NETs, which then block further development of *S. japonicum*. However, *S. japonicum* counteracts this response by upregulating host IL-10 expression. Our study enhances current understanding of the molecular mechanisms underlying *Schistosoma*-host interactions. Our data indicate that miR-142a-3p and WASL may be potential therapeutic targets for treating infectious diseases such as schistosomiasis and COVID-19.

## MATERIALS AND METHODS
### Animals and ethics
Male BALB/c mice (6 weeks old) were purchased from the Guangdong Medical Laboratory Animal Center, China. *WASL* gene KO mice (male, 6 weeks old, C57BL/6NCrl, Gene: WASL ENSMUSG00000031165) were provided by Professor Yinmin Liang of Xinxiang University. Mice were infected by percutaneous exposure to *S. japonicum* cercariae shed from lab-infected snails (*Oncomelania hupensis*), which were purchased from the National Institute of Parasitic Diseases, Chinese Center for Disease Control and Prevention. All procedures were approved by the Animal Care and Use Committee of Sun-Yat-Sen University (SYSU-IACUC-2020-B0063) and conformed to the Guidelines for the Care and Use of Laboratory Animals of the National Institute of Health in China.

### EV purification and identification
To prepare liver-derived EVs, the livers were collected from normal mice (n = 10) and infected mice (n = 10) at 56 days post-infection, and the gallbladder, bile ducts, and large vessels were removed. Single-cell (parenchymal cells and nonparenchymal cells) suspensions were obtained by gentle grinding. Single cells were resuspended in 90% Percoll and centrifuged at 1,000 $\times$ *g* for 5 min to remove dead-cell debris. The cells were maintained in RPMI-1640 culture medium ($1 \times 10^6$ cells/mL, total of 60 mL/mouse). After 24 h, the culture supernatant was collected for further experiments. EVs were purified by differential centrifugation and identified by electron microscopy, western blotting, and NTA. In brief, cell culture supernatants were centrifuged at low speed (300 $\times$ *g* for 10 min at 4°C) in 15-mL polypropylene tubes using a swinging-bucket rotor centrifuge (model A-4-44 5804R Refrigerated Centrifuge, Eppendorf, Germany), and the resulting supernatants were centrifuged at 2,000 $\times$ *g* for 10 min at 4°C. These supernatants were transferred into 1.5-mL polypropylene tubes (Eppendorf) using a micropipette and then centrifuged at 10,000 $\times$ *g* for 60 min at 4°C (fixed-angle rotor, angle of 45°, model no. 3331, D-37520 Refrigerated Centrifuge, Thermo Electron Corporation, USA). The resulting supernatants were transferred to Quick-Seal centrifuge tubes (Beckman Coulter, USA) and centrifuged at 120,000 $\times$ *g* for 90 min at 4°C in an Optima L-100xp ultracentrifuge (swinging-bucket rotor, model SW40 Ti, Optima L-100xp, Beckman Coulter). The resultant pellets (EVs) were diluted with PBS and centrifuged again at 120,000 $\times$ *g* for 90 min at 4°C in an Optima L-100xp ultracentrifuge (swinging-bucket rotor, model SW40 Ti, Optima L-100xp, Beckman Coulter). Negative-staining TEM was used to analyze the EVs. EVs were loaded on a copper grid and negatively stained with 3% (w/v) aqueous phosphotungstic acid for 1 min. The grid was then examined using a FEI Tecnai G2 Sprit Twin TEM (FEI, USA). Expression of the EV markers CD9, CD63, and CD81 was analyzed by western blotting. EV particles were also analyzed by NTA (NanoSight NS300, Malvern Instruments, UK) using a NanoSight NS300 instrument equipped with a sCMOS camera, 488-nm laser (blue), and NTA 3.3 Dev Build 3.3.30 software (number of frames: 749).

### Immunofluorescence analysis
Liver tissues were fixed in 4% neutral buffered formalin and embedded in paraffin. The paraffin sections were deparaffinized by baking and then dehydrated using xylene and ethanol. After blocking with 1% bovine serum albumin (BSA) for 1 h, the sections were incubated with anti-CD11b (1:100, BD Biosciences, USA), anti-Ly6G (1:100; BD Biosciences), anti-H3cit (1:400; CST, USA), anti-MPO

(1:100; Abcam, UK), and anti-N-WASP (1:100; Abcam) antibodies overnight at 4°C. Cells were fixed in 4% paraformaldehyde for 25 min at room temperature and then permeabilized with 0.2% Triton X-100 in PBS for 10 min. The cells were blocked in PBS with 2% BSA for 30 min at room temperature and then incubated with anti-H3Cit (1:400; CST), anti-N-WASP (1:100; Abcam), or anti-MPO (1:100; Abcam) antibodies overnight at 4°C. The sections and cells were then incubated with the indicated Alexa Fluor-conjugated secondary antibodies. DAPI was used to detect nuclei. Between all steps, samples were washed three times in PBS for 5 min each. The sections were visualized using an LSM 800 laser scanning confocal microscope (Zeiss, Germany).

#### Neutrophil and macrophage isolation

The femurs and tibia of mice were excised, and cells within the bone marrow were prepared as a single-cell suspension. Neutrophils were isolated from the single-cell suspension using Percoll density gradient centrifugation and identified and separated by positive selection for $CD11b^{+}Ly6G^{+}$ cells by flow cytometry (BD Influx, USA). For macrophage isolation, cells within the bone marrow were cultured in DMEM (Gibco, Germany) with 10% (v/v) fetal bovine serum and recombinant murine macrophage colony-stimulating factor (20 ng/mL, PeproTech, USA). On the seventh day, bone marrow-derived macrophages were harvested. Neutrophils and macrophages were cultured in a humidified, 5% $CO_2$ incubator at 37°C.

#### Analysis of EVs uptake

EVs were incubated with PKH26 (Sigma-Aldrich, USA) for 5 min, and staining was terminated by adding an equal volume of 1% BSA. The liquid was then transferred to a Quick-Seal centrifuge tube (Beckman Coulter) and centrifuged at 120,000 × *g* for 90 min at 4°C in an Optima L-100xp ultracentrifuge (swinging-bucket rotor, model SW60 Ti, Optima L-100xp, Beckman Coulter). The resultant pellet (PKH26-labeled EVs) was resuspended with PBS and centrifuged again at 120,000 × *g* for 90 min at 4°C in an Optima L-100xp ultracentrifuge (swinging-bucket rotor, model SW60 Ti, Optima L-100xp, Beckman Coulter). Neutrophils were incubated with PKH26-labeled EVs for 1 h, and the cells were analyzed using confocal microscopy to evaluate EV internalization. Alexa Fluor phalloidin-FITC (CST) was used to label actin, and DAPI was used to detect nuclei.

#### Scanning electron microscopy

Neutrophils were cultured on coverslips and treated as follows: PMA (500 nM; NET-inducer) for 4 h, NL-EVs (10 μg/mL) for 24 h; IL-EVs (10 μg/mL) for 24 h; *S. japonicum* SWAP (10 μg/mL) for 24 h; *S. japonicum* SEA (10 μg/mL) for 24 h; miR-142a-3p mimic (50 nM) for 24 h; Wiskostatin (N-WASP inhibitor, Abcam) (20 μM) for 24 h; or IL-10 (40 ng/mL) for 24 h. Neutrophils and *S. japonicum* worms were fixed in 2.5% glutaraldehyde overnight. After fixation, the samples were washed with PBS before dehydration using an ethanol gradient, followed by exchange of ethanol with acetone and isoamyl acetate. The samples were critical point-dried and coated with gold using an ion coater (E−102; Hitachi) and observed and photographed using an FEI Quanta 200 scanning electron microscope.

#### Small RNA sequencing and analysis

Small RNAs were sequenced and analyzed as described previously.[31] In brief, total RNA was isolated from EVs and used as the input material to generate the small RNA library. Following cluster generation, the library preparations were sequenced on an Illumina HiSeq 2500 platform (Illumina, USA). After sequencing, the data were subjected to preliminary analyses, including quality control analysis, comparative analysis, target gene functional annotation, quantification of miRNA expression levels, mRNA differential gene expression analysis, and Gene Ontology and Kyoto Encyclopedia of Genes and Genomes enrichment analyses.

#### RNA extraction and qRT-PCR

Expression of mRNAs was quantified using qRT-PCR. In brief, RNA was extracted from cells by lysing 100 mg of mouse liver tissue and cells ($10^6$ cells/sample) in TRIzol reagent (Invitrogen, USA) according to the manufacturer's instructions. The extracted RNA was then quantified using a NanoDrop ND-2000 spectrophotometer (Thermo Scientific, USA). Complementary DNA (cDNA) was synthesized from 1.0 μg of total RNA using oligo (dT) primers and a Thermo Scientific RevertAid First Strand cDNA synthesis kit (Thermo Scientific) according to the manufacturer's protocol. Expression of miR-142a-3p, WASL, IL-10, CCL2, α-SMA, collagen I, IL-1β, IL-23A, and IL-33 was analyzed using a SYBR Green Master Mix kit (Takara, Japan) with the primers listed in Table 1. GAPDH or U6 snRNA were used as internal control, and the fold-change in expression was calculated according to the $2^{-\Delta\Delta CT}$ method.

#### rAAV vectors and transduction

The rAAVs HBAAV2/9-miR-142a-3p-green fluorescent protein (GFP) and HBAAV2/9-GFP were purchased from Hanbio Biotechnology. Mice were injected via the tail vein with viral particles ($1.5 \times 10^{11}$ v.g./mouse) 3 days after infection with *S. japonicum*. GFP expression associated with HBAAV2/9 in the liver was observed using fluorescence microscopy.

#### Histopathology and fibrosis measurement

The indexes of the liver, spleen, and thymus were calculated based on the following formula: total weight of mouse liver or spleen or thymus (mg)/total weight of mouse (g). The area of egg granulomas was measured on H&E-stained tissue sections using Image Pro Plus 6.0 software. The area of each granuloma in H&E-stained sections was measured. Liver tissues were weighed and digested overnight in 4% potassium hydroxide, and released eggs were then counted. Worms were fixed with 95% ethanol, 3% formalin, and 2% glacial acetic acid and then stained with 2.5% hydrochloric carmine red (Merck) for 1 h and preserved on glass slides. The worms were observed under a stereomicroscope (Leica M205FA, Germany), and worm length was calculated using the stereomicroscope (Leica M205FA). Fibrosis was detected by staining liver sections with Masson's trichrome.

**Table 1. Primers used for qRT-PCR**

| Gene | Forward (5′-3′) | Reverse (5′-3′) |
|---|---|---|
| WASL | TCCTTCTAGGCCTGGTGTTG | CTGTGGACCCTGCCATAGAT |
| IL-10 | AGTACAGCCGGGAAGACAAT | TCTAGGAGCATGTGGCTCTG |
| CCL2 | AACTGCATCTGCCCTAAGGT | CTGTCACACTGGTCACTCCT |
| α-SMA | CACAGCCCTGGTGTGCGACAAT | TTGCTCTGGGCTTCATCCCCCA |
| Collagen I | TCCTGCGCCTAATGTCCACCGA | AAGCGACTGTTGCCTTCGCCTC |
| IL-1β | CTCACAAGCAGAGCACAAGC | TCCAGCCCATACTTTAGGAAGA |
| IL-23A | ATGCTGGATTGCAGAGCAGTA | ACGGGGCACATTATTTTTAGTCT |
| IL-33 | ACACAGTCTCCTGCCTCCCT | CCACACCGTCGCCTGATTGA |
| GAPDH (mouse) | ACTCCACTCACGGCAAATTC | TCTCCATGGTGGTGAAGACA |
| miR-142a-3p | CTGTAGTGTTTCCTACTTTATG | mRQ 3′ Primer (Takara, Kyoto, Japan) |
| U6 | Takara, Kyoto, Japan | Takara, Kyoto, Japan |

### Prediction of potential miR-142a-3p target genes and dual-luciferase reporter assay

TargetScan was used to predict potential miR-142a-3p target genes. Target genes were selected for further study by combining scores and conserved binding sites in TargetScan. HEK293T cells were transfected with reporter plasmids encoding the un-mutated or mutated 3′ UTR of WASL and with miR-142a-3p mimic or control mimic for 48 h using RNAiMAX (Invitrogen). Luciferase assay reagent (Promega, USA) was added to the cells, and luciferase activity was detected using an Infinite F500 Multimarker Analyzer (Tecan, Austria). Luciferase activity was normalized to *Renilla* luciferase activity.

### Western blotting

Liver tissues, neutrophils, and macrophages were homogenized using RIPA lysis buffer in the presence of freshly added phosphatase and protease inhibitors (Thermo Fisher Scientific, USA). Lysates were subjected to 10% sodium dodecyl sulfate-polyacrylamide gel electrophoresis, and the resolved proteins were transferred onto polyvinylidene fluoride blotting membranes (GE Healthcare Life Sciences, UK) and blocked using 5% skim milk. The membranes were incubated with the following primary antibodies (overnight at 4°C): anti-mouse N-WASP (1:1,000; Abcam), IL-10 (1:500; Proteintech, China), CCL2 (1:500; Boster, China), α-SMA (1:1,000; Abcam), collagen I (1:2,000; Proteintech), and TNF-α (1:1,000; Abcam). GAPDH (1:5,000; Sigma-Aldrich, USA) and β-actin (1:5,000; Sigma-Aldrich) were used as internal standards. Blots were incubated with anti-IgG conjugated to horseradish peroxidase as the secondary antibody for 2 h at room temperature. The membranes were visualized using an ECL western blotting detection system (Amersham, USA).

### Purification of NETs

Neutrophils were treated with 500 nM PMA for 4 h. The culture supernatant containing NETs was centrifuged at 1,000 × *g* for 10 min at 4°C, and isolated NETs were used for subsequent experiments.

### Statistical analysis

Results are expressed as mean ± SD. Differences between two groups were analyzed using unpaired two-sample t tests. Multiple comparisons between more than two groups were analyzed by one-way ANOVA. p values < 0.05 were considered statistically significant. Immunofluorescence and scanning electron microscopy data for NETs were quantified using ImageJ software. Statistical analyses were performed using GraphPad Prism 8.0.

## SUPPLEMENTAL INFORMATION

Supplemental information can be found online at https://doi.org/10.1016/j.ymthe.2022.03.016.

## ACKNOWLEDGMENTS

This work was supported by the National Key Research and Development Program of China (nos. 2021YFC2300800, 2021YFC2300801), the National Natural Science Foundation of China (grant nos. 81871682 and 81902081), the Natural Science Foundation of Guangdong Province (grant nos. 2020A1515011573, 2019A1515012068, and 2021A1515010976), and the Guangzhou Science and Technology Program (grant no. 202102100003). Generation of *WASL* gene knockout mice in the C57BL/6 background was supported by the 111 program (D20036).

## AUTHOR CONTRIBUTIONS

L.W., X.S., and Z.W. conceived and designed the experiments. L.W., Z.Y., and Y.L. acquired the data. Z.Y. performed and analyzed the data for most of the experiments. L.Z., R.Y., and J.L. assisted with performing experiments and analyzing data. L.W., X.S., and Z.W. wrote the manuscript.

## DECLARATION OF INTERESTS

The authors declare no competing interests.

## REFERENCES

1. Wendt, G., Zhao, L., Chen, R., Liu, C., O'Donoghue, A.J., Caffrey, C.R., Reese, M.L., and Collins, J.J. (2020). A single-cell RNA-seq atlas of Schistosoma mansoni identifies

a key regulator of blood feeding. Science *369*, 1644–1649. https://doi.org/10.1126/science.abb7709.

2. Liu, S., Zhang, X., Liu, Y., Yuan, X., Yang, L., Zhang, R., Wang, X., Xu, F., and Zhu, C. (2020). Early application of caffeine improves white matter development in very preterm infants. Respir. Physiol. Neurobiol. *281*, 103495. https://doi.org/10.1016/j.resp.2020.103495.
3. Chuah, C., Jones, M.K., Burke, M.L., McManus, D.P., and Gobert, G.N. (2014). Cellular and chemokine-mediated regulation in schistosome-induced hepatic pathology. Trends Parasitol. *30*, 141–150. https://doi.org/10.1016/j.pt.2013.12.009.
4. Saitoh, T., Komano, J., Saitoh, Y., Misawa, T., Takahama, M., Kozaki, T., Uehata, T., Iwasaki, H., Omori, H., Yamaoka, S., et al. (2012). Neutrophil extracellular traps mediate a host defense response to human immunodeficiency virus-1. Cell Host Microbe *12*, 109–116. https://doi.org/10.1016/j.chom.2012.05.015.
5. Burke, M.L., McManus, D.P., Ramm, G.A., Duke, M., Li, Y., Jones, M.K., and Gobert, G.N. (2010). Temporal expression of chemokines dictates the hepatic inflammatory infiltrate in a murine model of schistosomiasis. PLoS Negl. Trop. Dis. *4*, e598. https://doi.org/10.1371/journal.pntd.0000598.
6. Wu, C., Chen, Q., Fang, Y., Wu, J., Han, Y., Wang, Y., Yang, Y., Chu, M., Feng, Y., Tan, L., et al. (2014). *Schistosoma japonicum* egg specific protein SjE16.7 recruits neutrophils and induces inflammatory hepatic granuloma initiation. PLos Negl. Trop. Dis. *8*, e2703. https://doi.org/10.1371/journal.pntd.0002703.
7. Hirata, M., Kage, M., Hara, T., Yoneda, Y., Zhang, M., and Fukuma, T. (2001). *Schistosoma japonicum* egg granuloma formation in the interleukin-4 or interferon-gamma deficient host. Parasite Immunol. *23*, 271–280. https://doi.org/10.1046/j.1365-3024.2001.00382.x.
8. van Kessel, K.P.M., Bestebroer, J., and van Strijp, J.A.G. (2014). Neutrophil-mediated phagocytosis of Staphylococcus aureus. Front. Immunol. *5*, 467. https://doi.org/10.3389/fimmu.2014.00467.
9. Swamydas, M., Gao, J.-L., Break, T.J., Johnson, M.D., Jaeger, M., Rodriguez, C.A., Lim, J.K., Green, N.M., Collar, A.L., Fischer, B.G., et al. (2016). CXCR1-mediated neutrophil degranulation and fungal killing promote Candida clearance and host survival. Sci. Transl. Med. *8*, 322ra310. https://doi.org/10.1126/scitranslmed.aac7718.
10. Papayannopoulos, V. (2018). Neutrophil extracellular traps in immunity and disease. Nat. Rev. Immunol. *18*, 134–147. https://doi.org/10.1038/nri.2017.105.
11. Brinkmann, V., Reichard, U., Goosmann, C., Fauler, B., Uhlemann, Y., Weiss, D.S., Weinrauch, Y., and Zychlinsky, A. (2004). Neutrophil extracellular traps kill bacteria. Science (New York, N.Y.) *303*, 1532–1535. https://doi.org/10.1126/science.1092385.
12. Sollberger, G., Choidas, A., Burn, G.L., Habenberger, P., Di Lucrezia, R., Kordes, S., Menninger, S., Eickhoff, J., Nussbaumer, P., Klebl, B., et al. (2018). Gasdermin D plays a vital role in the generation of neutrophil extracellular traps. Sci. Immunol. *3*, eaar6689. https://doi.org/10.1126/sciimmunol.aar6689.
13. Urban, C.F., Reichard, U., Brinkmann, V., and Zychlinsky, A. (2006). Neutrophil extracellular traps capture and kill *Candida albicans* yeast and hyphal forms. Cell Microbiol. *8*, 668–676. https://doi.org/10.1111/j.1462-5822.2005.00659.x.
14. Cicco, S., Cicco, G., Racanelli, V., and Vacca, A. (2020). Neutrophil extracellular traps (NETs) and damage-associated molecular patterns (DAMPs): two potential targets for COVID-19 treatment. Mediators Inflamm. *2020*, 1–25. https://doi.org/10.1155/2020/7527953.
15. Abi Abdallah, D.S., Lin, C., Ball, C.J., King, M.R., Duhamel, G.E., and Denkers, E.Y. (2012). *Toxoplasma gondii* triggers release of human and mouse neutrophil extracellular traps. Infect. Immun. *80*, 768–777. https://doi.org/10.1128/iai.05730-11.
16. Knackstedt, S.L., Georgiadou, A., Apel, F., Abu-Abed, U., Moxon, C.A., Cunnington, A.J., Raupach, B., Cunningham, D., Langhorne, J., Kruger, R., et al. (2019). Neutrophil extracellular traps drive inflammatory pathogenesis in malaria. Sci. Immunol. *4*, eaaw0336. https://doi.org/10.1126/sciimmunol.aaw0336.
17. Gabriel, C., McMaster, W.R., Girard, D., and Descoteaux, A. (2010). *Leishmania donovani* promastigotes evade the antimicrobial activity of neutrophil extracellular traps. J. Immunol. *185*, 4319–4327. Baltimore, Md, 1950. https://doi.org/10.4049/jimmunol.1000893.
18. Bonne-Année, S., Kerepesi, L.A., Hess, J.A., Wesolowski, J., Paumet, F., Lok, J.B., Nolan, T.J., and Abraham, D. (2014). Extracellular traps are associated with human and mouse neutrophil and macrophage mediated killing of larval *Strongyloides stercoralis*. Microbes Infect. *16*, 502–511. https://doi.org/10.1016/j.micinf.2014.02.012.
19. Bouchery, T., Moyat, M., Sotillo, J., Silverstein, S., Volpe, B., Coakley, G., Tsourouktsoglou, T.D., Becker, L., Shah, K., Kulagin, M., et al. (2020). Hookworms evade host immunity by secreting a deoxyribonuclease to degrade neutrophil extracellular traps. Cell Host Microbe *27*, 277–289.e6. https://doi.org/10.1016/j.chom.2020.01.011.
20. Chuah, C., Jones, M.K., Burke, M.L., Owen, H.C., Anthony, B.J., McManus, D.P., Ramm, G.A., and Gobert, G.N. (2013). Spatial and temporal transcriptomics of *Schistosoma japonicum*-induced hepatic granuloma formation reveals novel roles for neutrophils. J. Leukoc. Biol. *94*, 353–365. https://doi.org/10.1189/jlb.1212653.
21. Lőrincz, Á.M., Bartos, B., Szombath, D., Szeifert, V., Timár, C.I., Turiák, L., Drahos, L., Kittel, A., Veres, D.S., Kolonics, F., et al. (2020). Role of Mac-1 integrin in generation of extracellular vesicles with antibacterial capacity from neutrophilic granulocytes. J. Extracell. Vesicles *9*, 1698889. https://doi.org/10.1080/20013078.2019.1698889.
22. Angioni, R., Liboni, C., Herkenne, S., Sánchez-Rodríguez, R., Borile, G., Marcuzzi, E., Cali, B., Muraca, M., and Viola, A. (2020). CD73+extracellular vesicles inhibit angiogenesis through adenosine A2Breceptor signalling. J. Extracell. Vesicles *9*, 1757900. https://doi.org/10.1080/20013078.2020.1757900.
23. Cavallaro, S., Pevere, F., Stridfeldt, F., Görgens, A., Paba, C., Sahu, S.S., Mamand, D.R., Gupta, D., El Andaloussi, S., Linnros, J., et al. (2021). Multiparametric profiling of single nanoscale extracellular vesicles by combined atomic force and fluorescence microscopy: correlation and heterogeneity in their molecular and biophysical features. Small *17*, e2008155. https://doi.org/10.1002/smll.202008155.
24. Fan, Z., Xiao, K., Lin, J., Liao, Y., and Huang, X. (2019). Functionalized DNA enables programming exosomes/vesicles for tumor imaging and therapy. Small *15*, e1903761. https://doi.org/10.1002/smll.201903761.
25. Benmoussa, A., Ly, S., Shan, S.T., Laugier, J., Boilard, E., Gilbert, C., and Provost, P. (2017). A subset of extracellular vesicles carries the bulk of microRNAs in commercial dairy cow's milk. J. Extracell. Vesicles *6*, 1401897. https://doi.org/10.1080/20013078.2017.1401897.
26. Min, L., Zhu, S., Chen, L., Liu, X., Wei, R., Zhao, L., Yang, Y., Zhang, Z., Kong, G., Li, P., and Zhang, S. (2019). Evaluation of circulating small extracellular vesicles derived miRNAs as biomarkers of early colon cancer: a comparison with plasma total miRNAs. J. Extracell. Vesicles *8*, 1643670. https://doi.org/10.1080/20013078.2019.1643670.
27. Xiong, Y., Chen, L., Yan, C., Zhou, W., Endo, Y., Liu, J., Hu, L., Hu, Y., Mi, B., and Liu, G. (2020). Circulating exosomal miR-20b-5p inhibition restores Wnt9b signaling and reverses diabetes-associated impaired wound healing. Small *16*, e1904044. https://doi.org/10.1002/smll.201904044.
28. Zhang, L., Hao, C., Yao, S., Tang, R., Guo, W., Cong, H., Li, J., Bao, L., Wang, D., Li, Y., et al. (2018). Exosomal miRNA profiling to identify nanoparticle phagocytic mechanisms. Small *14*, e1704008. https://doi.org/10.1002/smll.201704008.
29. Zhu, S., Wang, S., Lin, Y., Jiang, P., Cui, X., Wang, X., Zhang, Y., and Pan, W. (2016). Release of extracellular vesicles containing small RNAs from the eggs of *Schistosoma japonicum*. Parasites Vectors *9*, 574. https://doi.org/10.1186/s13071-016-1845-2.
30. Meningher, T., Barsheshet, Y., Ofir-Birin, Y., Gold, D., Brant, B., Dekel, E., Sidi, Y., Schwartz, E., Regev-Rudzki, N., Avni, O., and Avni, D. (2020). Schistosomal extracellular vesicle-enclosed miRNAs modulate host T helper cell differentiation. EMBO Rep. *21*, e47882. https://doi.org/10.15252/embr.201947882.
31. Wang, L., Liao, Y., Yang, R., Yu, Z., Zhang, L., Zhu, Z., Wu, X., Shen, J., Liu, J., Xu, L., et al. (2020). Sja-miR-71a in Schistosome egg-derived extracellular vesicles suppresses liver fibrosis caused by schistosomiasis via targeting semaphorin 4D. J. Extracell. Vesicles *9*, 1785738. https://doi.org/10.1080/20013078.2020.1785738.
32. Mori, M.A., Ludwig, R.G., Garcia-Martin, R., Brandão, B.B., and Kahn, C.R. (2019). Extracellular miRNAs: from biomarkers to mediators of physiology and disease. Cell Metab. *30*, 656–673. https://doi.org/10.1016/j.cmet.2019.07.011.
33. Coakley, G., and Harris, N.L. (2020). Interactions between macrophages and helminths. Parasite Immunol. *42*, e12717. https://doi.org/10.1111/pim.12717.
34. Gryseels, B., Polman, K., Clerinx, J., and Kestens, L. (2006). Human schistosomiasis. Lancet (London, England) *368*, 1106–1118. https://doi.org/10.1016/s0140-6736(06)69440-3.

35. McManus, D.P., Dunne, D.W., Sacko, M., Utzinger, J., Vennervald, B.J., and Zhou, X.-N. (2018). Schistosomiasis. Nat. Rev. Dis. Primers *4*, 13. https://doi.org/10.1038/s41572-018-0013-8.

36. Li Hsü, S.Y., Hsü, H.F., Davis, J.R., and Lust, G.L. (1972). Comparative studies on the lesions caused by eggs of *Schistosoma japonicum* and *Schistosoma mansoni* in livers of albino mice and rhesus monkeys. Ann. Trop. Med. Parasitol. *66*, 89–97. https://doi.org/10.1080/00034983.1972.11686801.

37. Hirata, M., and Fukuma, T. (2003). Cytokine regulation in experimentally-induced *Schistosoma japonicum* egg granuloma formation. Parasitol. Int. *52*, 341–349. https://doi.org/10.1016/s1383-5769(03)00050-3.

38. Chen, K.W., Monteleone, M., Boucher, D., Sollberger, G., Ramnath, D., Condon, N.D., von Pein, J.B., Broz, P., Sweet, M.J., and Schroder, K. (2018). Noncanonical inflammasome signaling elicits gasdermin D-dependent neutrophil extracellular traps. Sci. Immunol. *3*, eaar6676. https://doi.org/10.1126/sciimmunol.aar6676.

39. Bautista-Becerril, B., Campi-Caballero, R., Sevilla-Fuentes, S., Hernández-Regino, L.M., Hanono, A., Flores-Bustamante, A., Gonzalez-Flores, J., Garcia-Avila, C.A., Aquino-Galvez, A., Castillejos-Lopez, M., et al. (2021). Immunothrombosis in COVID-19: implications of neutrophil extracellular traps. Biomolecules *11*, 694. https://doi.org/10.3390/biom11050694.

40. Veras, F.P., Pontelli, M.C., Silva, C.M., Toller-Kawahisa, J.E., de Lima, M., Nascimento, D.C., Schneider, A.H., Caetite, D., Tavares, L.A., Paiva, I.M., et al. (2020). SARS-CoV-2-triggered neutrophil extracellular traps mediate COVID-19 pathology. J. Exp. Med. *217*, e20201129. https://doi.org/10.1084/jem.20201129.

41. Middleton, E.A., He, X.-Y., Denorme, F., Campbell, R.A., Ng, D., Salvatore, S.P., Mostyka, M., Baxter-Stoltzfus, A., Borczuk, A.C., Loda, M., et al. (2020). Neutrophil extracellular traps contribute to immunothrombosis in COVID-19 acute respiratory distress syndrome. Blood *136*, 1169–1179. https://doi.org/10.1182/blood.2020007008.

42. Barnes, B.J., Adrover, J.M., Baxter-Stoltzfus, A., Borczuk, A., Cools-Lartigue, J., Crawford, J.M., DaBler-Plenker, J., Guerci, P., Huynh, C., Knight, J.S., et al. (2020). Targeting potential drivers of COVID-19: neutrophil extracellular traps. J. Exp. Med. *217*, e20200652. https://doi.org/10.1084/jem.20200652.

43. Kho, S., Minigo, G., Andries, B., Leonardo, L., Prayoga, P., Poespoprodjo, J.R., Kenangalem, E., Price, R.N., Woodberry, T., Anstey, N.M., and Yeo, T.W. (2019). Circulating neutrophil extracellular traps and neutrophil activation are increased in proportion to disease severity in human malaria. J. Infect. Dis. *219*, 1994–2004. https://doi.org/10.1093/infdis/jiy661.

44. Muñoz-Caro, T., Rubio, R.M.C., Silva, L.M.R., Magdowski, G., Gärtner, U., McNeilly, T.N., Taubert, A., and Hermosilla, C. (2015). Leucocyte-derived extracellular trap formation significantly contributes to *Haemonchus contortus* larval entrapment. Parasites Vectors *8*, 607. https://doi.org/10.1186/s13071-015-1219-1.

45. Mathieu, M., Martin-Jaular, L., Lavieu, G., and Théry, C. (2019). Specificities of secretion and uptake of exosomes and other extracellular vesicles for cell-to-cell communication. Nat. Cel. Biol. *21*, 9–17. https://doi.org/10.1038/s41556-018-0250-9.

46. Ren, X.-S., Tong, Y., Qiu, Y., Ye, C., Wu, N., Xiong, X.-Q., Wang, J., Han, Y., Zhou, Y., Zhang, F., et al. (2020). MiR155-5p in adventitial fibroblasts-derived extracellular vesicles inhibits vascular smooth muscle cell proliferation via suppressing angiotensin-converting enzyme expression. J. Extracell. Vesicles *9*, 1698795. https://doi.org/10.1080/20013078.2019.1698795.

47. Chen, C.-Z., Li, L., Lodish, H.F., and Bartel, D.P. (2004). MicroRNAs modulate hematopoietic lineage differentiation. Science (New York, N.Y.) *303*, 83–86. https://doi.org/10.1126/science.1091903.

48. Nimmo, R., Ciau-Uitz, A., Ruiz-Herguido, C., Soneji, S., Bigas, A., Patient, R., and Enver, T. (2013). MiR-142-3p controls the specification of definitive hemangioblasts during ontogeny. Dev. Cell *26*, 237–249. https://doi.org/10.1016/j.devcel.2013.06.023.

49. Zhou, Q., Haupt, S., Prots, I., Thümmler, K., Kremmer, E., Lipsky, P.E., Schulze-Koops, H., and Skapenko, A. (2013). miR-142-3p is involved in CD25+ CD4 T cell proliferation by targeting the expression of glycoprotein A repetitions predominant. J. Immunol. *190*, 6579–6588. Baltimore, Md, 1950. https://doi.org/10.4049/jimmunol.1202993.

50. Lu, X., Li, X., He, Q., Gao, J., Gao, Y., Liu, B., and Liu, F. (2013). miR-142-3p regulates the formation and differentiation of hematopoietic stem cells in vertebrates. Cell Res. *23*, 1356–1368. https://doi.org/10.1038/cr.2013.145.

51. Fan, H.-B., Liu, Y.-J., Wang, L., Du, T.-T., Dong, M., Gao, L., Meng, Z.Z., Jin, Y., Chen, Y., Deng, M., et al. (2014). miR-142-3p acts as an essential modulator of neutrophil development in zebrafish. Blood *124*, 1320–1330. https://doi.org/10.1182/blood-2013-12-545012.

52. Westerberg, L.S., Dahlberg, C., Baptista, M., Moran, C.J., Detre, C., Keszei, M., Eston, M.A., Alt, F.W., Terhorst, C., Notarangelo, L.D., and Snapper, S.B. (2012). Wiskott-Aldrich syndrome protein (WASP) and N-WASP are critical for peripheral B-cell development and function. Blood *119*, 3966–3974. https://doi.org/10.1182/blood-2010-09-308197.

53. Volpi, S., Santori, E., Abernethy, K., Mizui, M., Dahlberg, C.I.M., Recher, M., Capuder, K., Csizmadia, E., Ryan, D., Mathew, D., et al. (2016). N-WASP is required for B-cell-mediated autoimmunity in Wiskott-Aldrich syndrome. Blood *127*, 216–220. https://doi.org/10.1182/blood-2015-05-643817.

54. Dahlberg, C.I.M., Torres, M.-L., Petersen, S.H., Baptista, M.A.P., Keszei, M., Volpi, S., Grasset, E.K., Karlsson, M.C., Walter, J.E., Snapper, S.B., et al. (2015). Deletion of WASp and N-WASp in B cells cripples the germinal center response and results in production of IgM autoantibodies. J. Autoimmun. *62*, 81–92. https://doi.org/10.1016/j.jaut.2015.06.003.

55. Qian, B.-Z., Li, J., Zhang, H., Kitamura, T., Zhang, J., Campion, L.R., Kaiser, E.A., Snyder, L.A., and Pollard, J.W. (2011). CCL2 recruits inflammatory monocytes to facilitate breast-tumour metastasis. Nature *475*, 222–225. https://doi.org/10.1038/nature10138.

56. He, X., Sun, Y., Lei, N., Fan, X., Zhang, C., Wang, Y., Zheng, K., Zhang, D., and Pan, W. (2018). *MicroRNA-351* promotes schistosomiasis-induced hepatic fibrosis by targeting the vitamin D receptor. Proc. Natl. Acad. Sci. U S A. *115*, 180–185. https://doi.org/10.1073/pnas.1715965115.

57. Wiedemann, M., and Voehringer, D. (2020). Immunomodulation and immune escape strategies of gastrointestinal helminths and schistosomes. Front. Immunol. *11*, 572865. https://doi.org/10.3389/fimmu.2020.572865.

58. Allen, J.E., and Maizels, R.M. (2011). Diversity and dialogue in immunity to helminths. Nat. Rev. Immunol. *11*, 375–388. https://doi.org/10.1038/nri2992.

59. Sun, X., Yang, F., Shen, J., Liu, Z., Liang, J., Zheng, H., Fung, M., and Wu, Z. (2016). Recombinant Sj16 from *Schistosoma japonicum* contains a functional N-terminal nuclear localization signal necessary for nuclear translocation in dendritic cells and interleukin-10 production. Parasitol. Res. *115*, 4559–4571. https://doi.org/10.1007/s00436-016-5247-3.

**YMTHE, Volume *30***

## Supplemental Information

# Host liver-derived extracellular vesicles deliver miR-142a-3p induces neutrophil extracellular traps via targeting WASL to block the development of *Schistosoma japonicum*

**Lifu Wang, Zifeng Zhu, Yao Liao, Lichao Zhang, Zilong Yu, Ruibing Yang, Ji Wu, Zhongdao Wu, and Xi Sun**

## Supplemental Information

# Host Liver-Derived Extracellular Vesicles Deliver miR-142a-3p Induces Neutrophil Extracellular Traps via Targeting WASL to Block the Development of *Schistosoma japonicum*

Lifu Wang, Zifeng Zhu, Yao Liao, Lichao Zhang, Zilong Yu, Ruibing Yang, Ji Wu, Zhongdao Wu, and Xi Sun

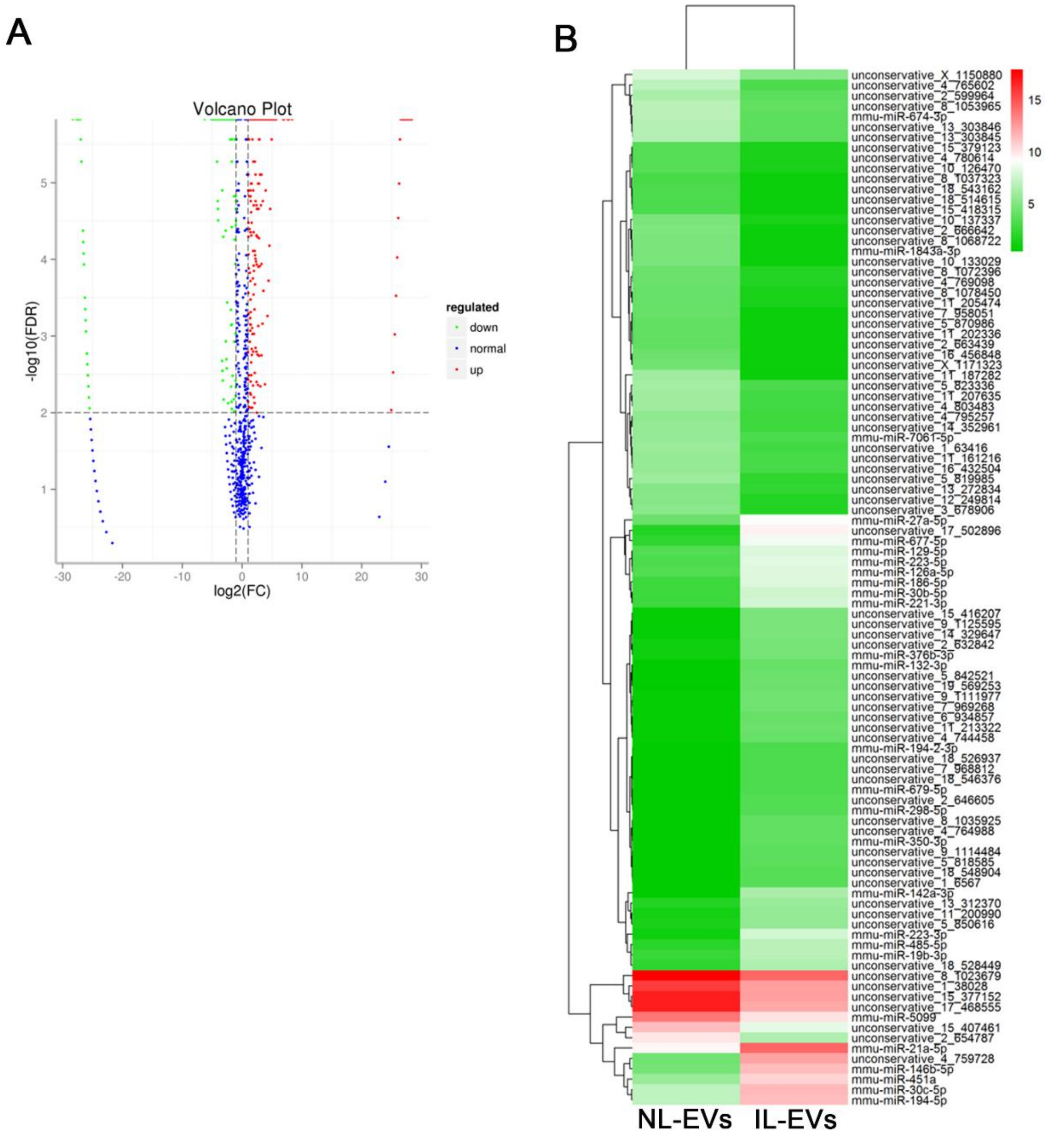


**Figure S1. miRNAs of EVs derived from liver of *S. japonicum*–infected mice (IL-**

**EVs) and normal liver–derived EVs (IL-EVs) were sequenced and analyzed. (A)** Volcano diagrams of miRNAs derived from the comparative gene expression analyses. Red dots represent up-regulated miRNAs, and green dots represent down-regulated miRNAs. **(B)** Heat map analysis shows differentially expressed miRNAs. Bright green=low expression; bright red=high expression.

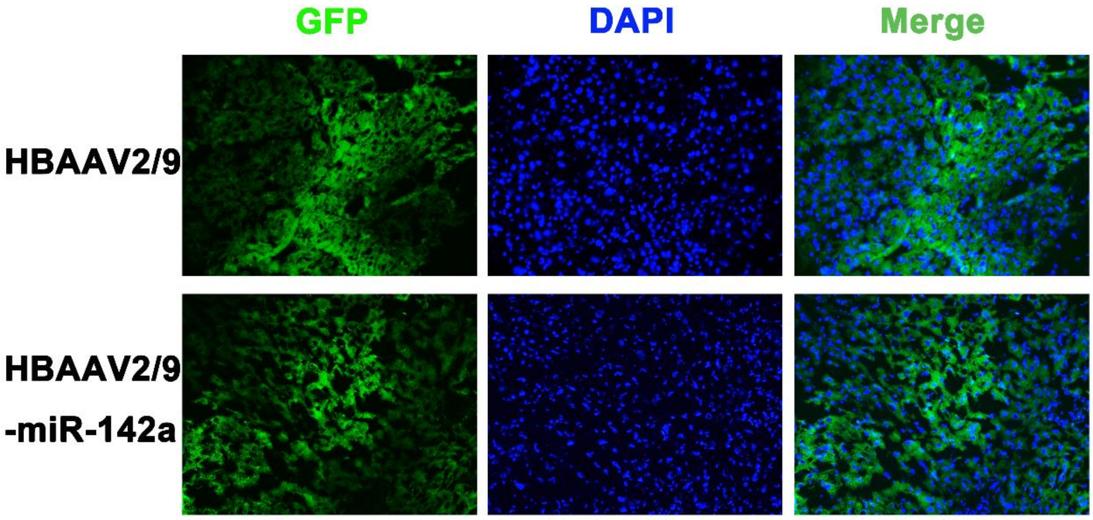


**Figure S2. HBAAV2/9-miR-142a-3p was constructed and successfully transfected into mice (HBAAV2/9 expresses GFP).** HBAAV2/9-miR-142a-3p colonization in the liver was analyzed by fluorescence microscopy.

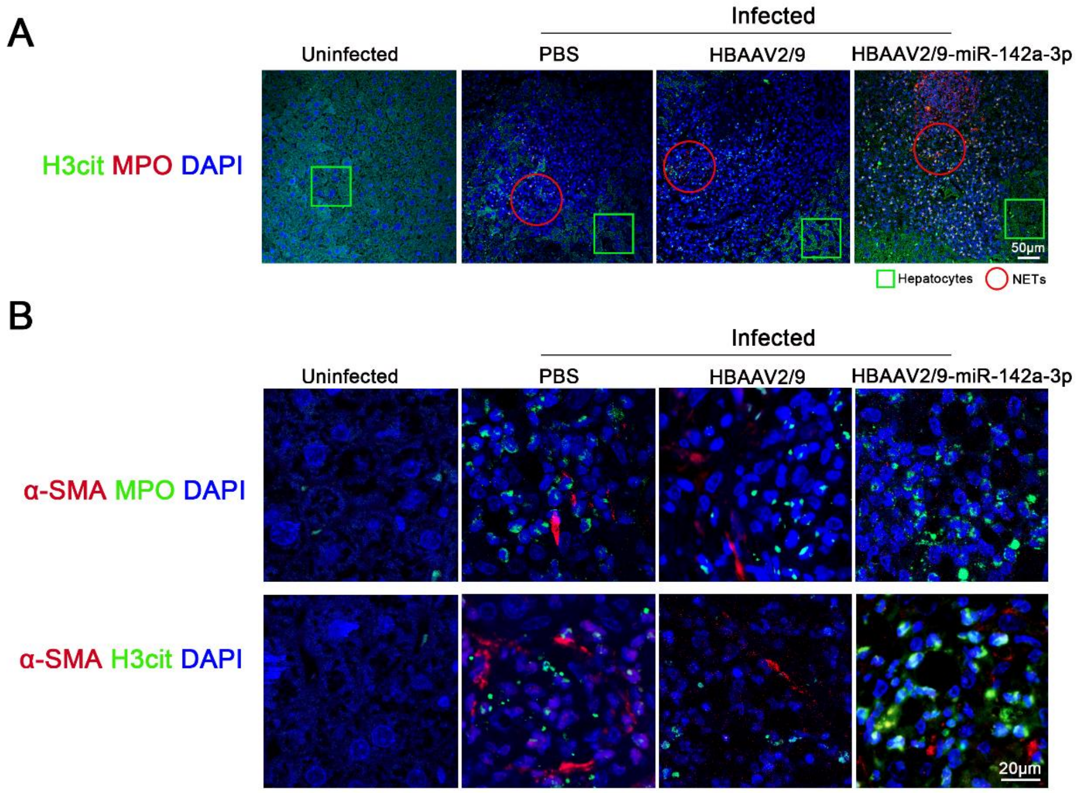


**Figure S3. NETs were observed in the granulomas of *S. japonicum*. (A)** Location of NETs in liver sections was observed based on H3cit and MPO co-localization. **(B)** Co-localization of H3cit, MPO, and α-SMA was observed by immunofluorescence.

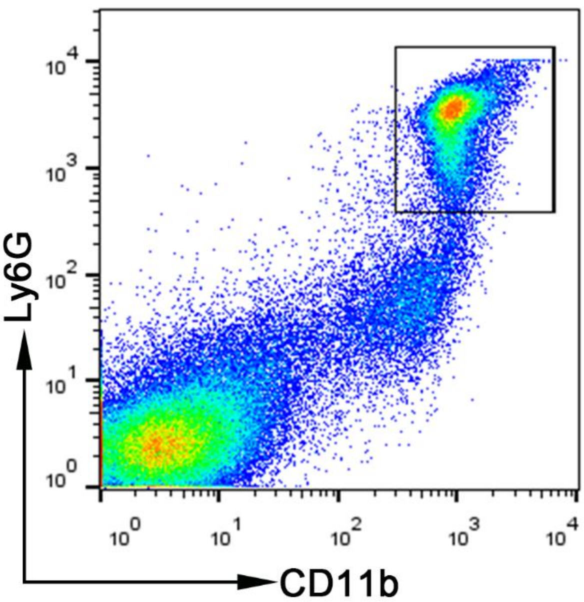


**Figure S4. Neutrophils were isolated using Percoll density gradient centrifugation and flow cytometry.**

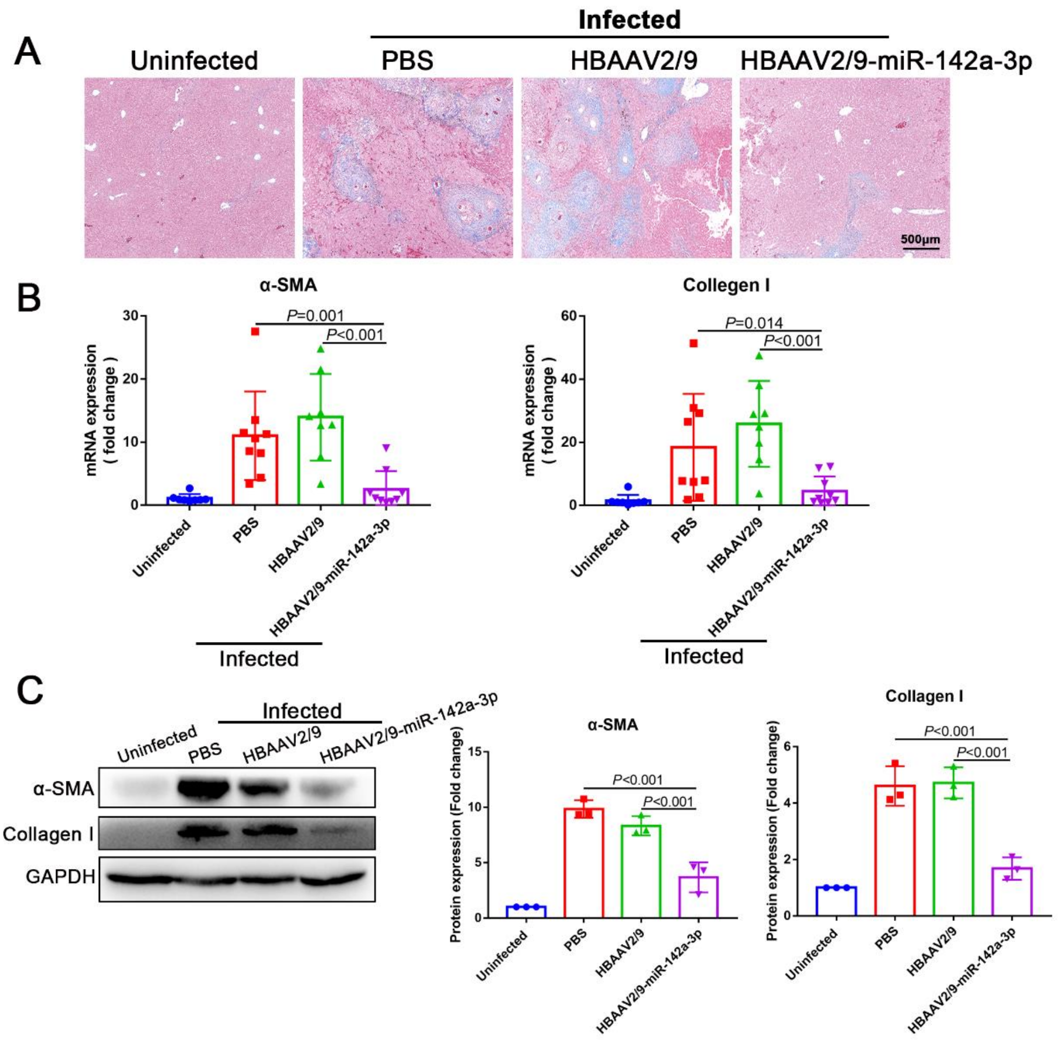


**Figure S5. miR-142a-3p attenuated liver fibrosis in *S. japonicum* infection. (A)** Fibrosis was observed in liver sections stained with Masson's trichrome. **(B)** Expression levels of α-SMA and collagen I in mouse liver were analyzed by qRT-PCR (n=8-9 mice per group). **(C)** Expression levels of α-SMA and collagen I in mouse liver were analyzed by Western blotting. Results are shown as mean ± SD (one-way ANOVA with Dunnett's multiple comparison test).

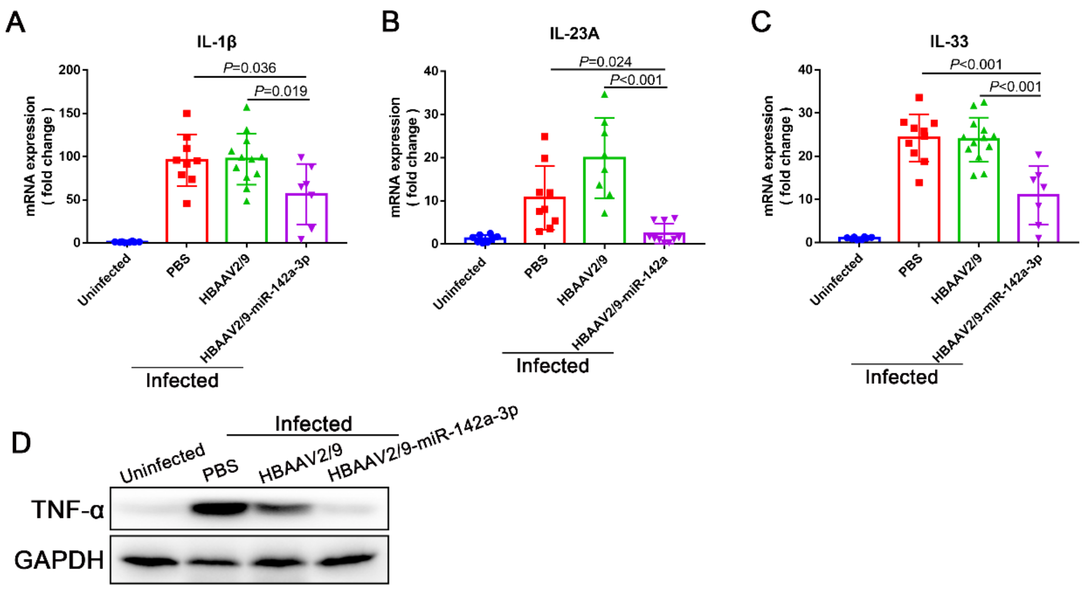


**Figure S6.** High expression of the inflammatory cytokines IL-1β **(A)**, IL-23A **(B)**, IL-33 **(C)**, and TNF-α **(D)** in liver tissues during *S. japonicum* infection decreased significantly after HBAAV2/9-miR-142a-3p treatment (n=7-12 mice per group).

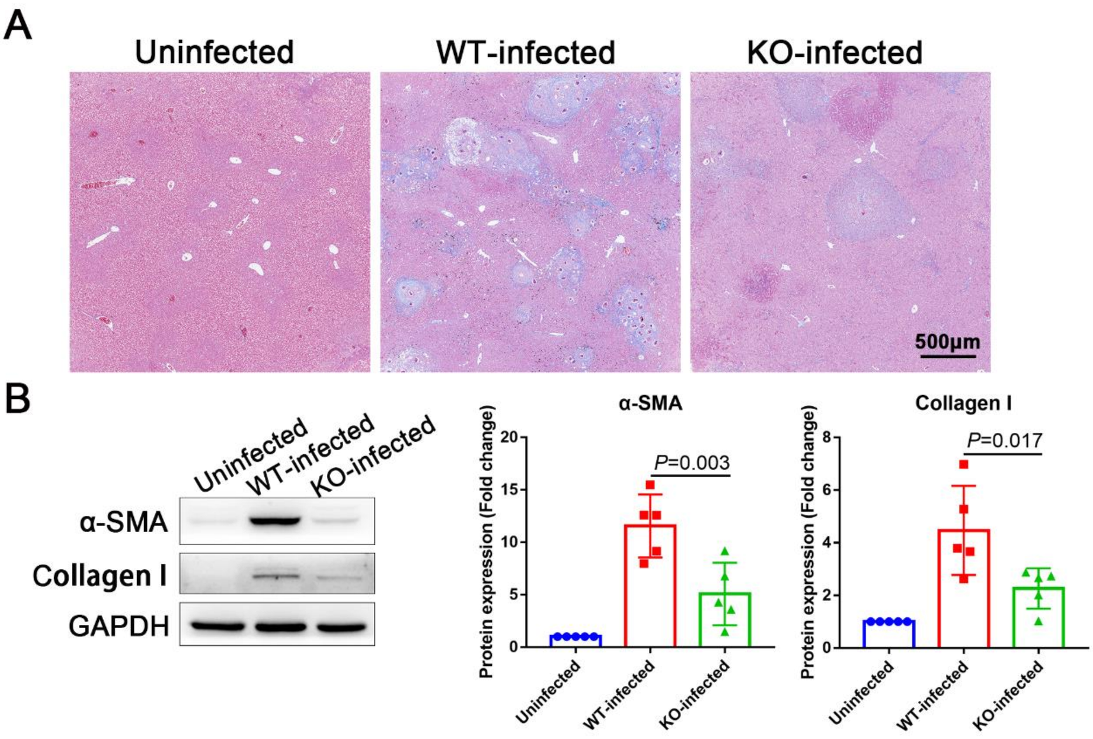


**Figure S7. WASL-deletion attenuated liver fibrosis in *S. japonicum* infection. (A)** Fibrosis was observed in liver sections stained with Masson's trichrome. **(B)**

Expression levels of α-SMA and collagen I in mouse liver were analyzed by Western blotting. Results are shown as mean ± SD (one-way ANOVA with Dunnett's multiple comparison test).

**Table S1. Particle characteristics and yield of NL-EVs and IL-EVs**

| | NL-EVs | IL-EVs |
|---|---|---|
| Mice | n=10 | n=10 |
| Cell culture time of liver tissues | 24 h | 24 h |
| Cell density (cells/mL) | $1\times10^6$ | $1\times10^6$ |
| Particle concentration (particles/mL) | $9.72\times10^{10}$ | $6.69\times10^{10}$ |
| Protein concentration (mg/mL) | 7.34 | 5.21 |
| Total volume of EVs (in PBS) | 300 μL | 400 μL |

**Table S2. The top ten differentially-expressed miRNA (IL-EVs/NL-EVs).**

| NO. | miRNA | log2FC | regulated |
|---|---|---|---|
| 1 | mmu-miR-142a-3p | 7.753542806 | Up |
| 2 | mmu-miR-223-3p | 7.615122837 | Up |
| 3 | mmu-miR-677-5p | 7.006138383 | Up |
| 4 | mmu-miR-146b-5p | 6.866236731 | Up |
| 5 | mmu-miR-186-5p | 5.572362953 | Up |
| 6 | mmu-miR-132-3p | 5.49367909 | Up |
| 7 | mmu-miR-350-3p | 5.333210545 | Up |

| | | | |
|---|---|---|---|
| 8 | mmu-miR-485-5p | 5.322722633 | Up |
| 9 | mmu-miR-30b-5p | 5.315091289 | Up |
| 10 | mmu-miR-221-3p | 5.176670648 | Up |

**WASL gene knockout mice**

Gene: ENSMUSG00000031165

Description: Wiskott-Aldrich syndrome [Source:MGI Symbol;Acc:MGI:105059]

Location: Chromosome X: 7,947,692-7,956,737 reverse strand.

Primers' sequences: "GGCCCTGGAGGACTTATTTC" and "AGCTCAGGGGGTCACTGATA"

PCR products: WT (811 bp) and KO (~200 bp)

| PCR Reaction | |
|---|---|
| 2×Taq Master Mix | 7.5 µL |
| Forward primers (10 µM) | 0.3 µL |
| Reverse primers (10 µM) | 0.3 µL |
| Genomic DNA (20 ng/µL) | 1.0 µL |
| Add H2O up to | 15 µL |

| PCR Program | | |
|---|---|---|
| | 94 °C | 5 min |
| 35 cycles | 94 °C | 30 sec |
| | 60 °C | 30 sec |
| | 72 °C | 70 sec |
| | 72 °C | 10 min |

**PCR products: WT (811 bp) and KO (~200 bp)**

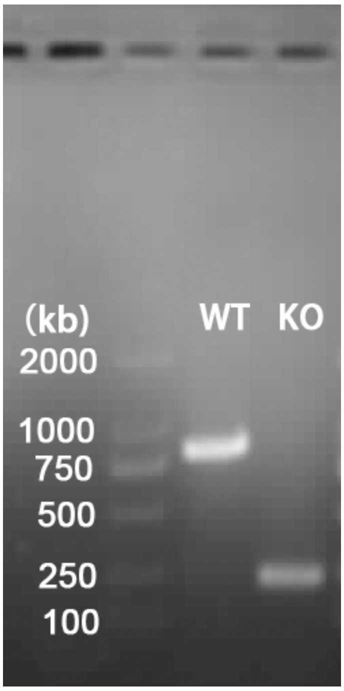
(kb)
WT
KO
2000
1000
750
500
250
100

**Negative control for immunofluorescence**

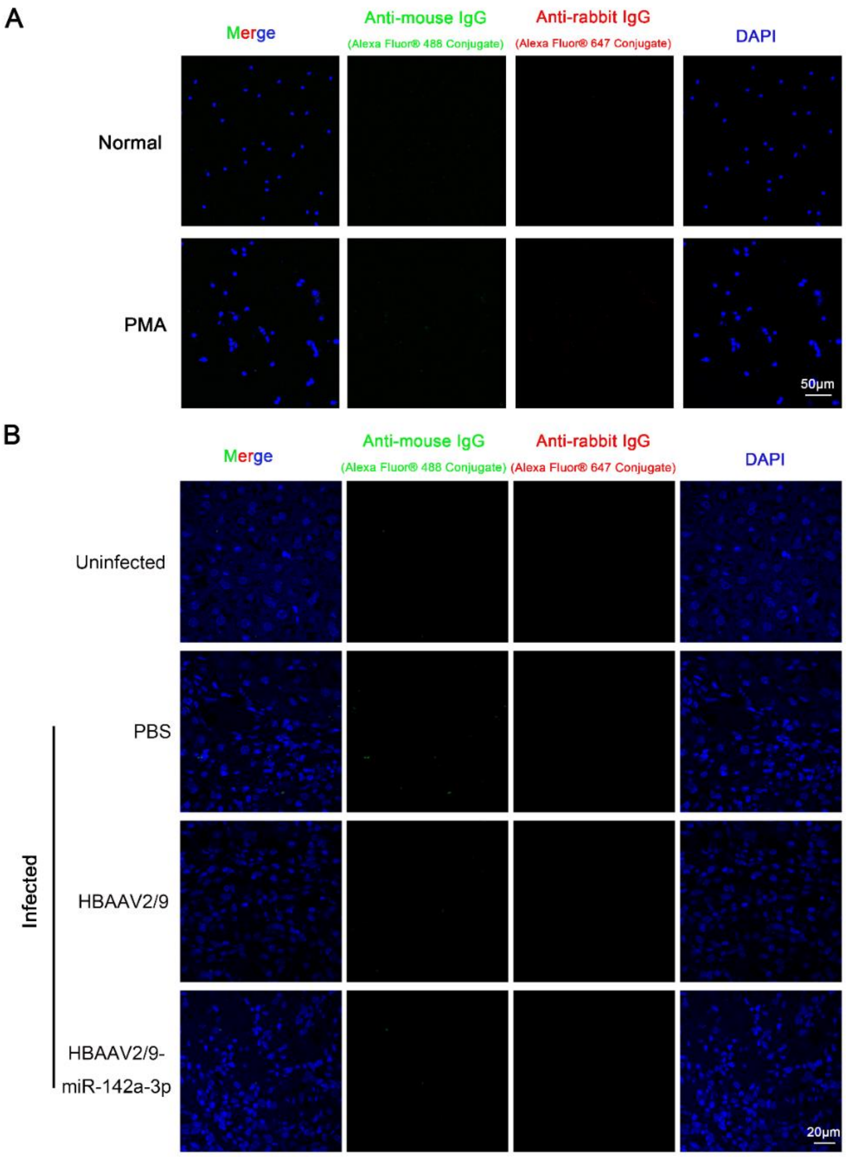


**Negative control for immunofluorescence**

**Figure (A)** Neutrophils were treated with PMA (500 nM, 4 h), and the non-specific staining effect of the secondary antibody was excluded by direct use of secondary antibody without adding primary antibody. **(B)** The non-specific staining effect of the secondary antibody on liver slices was excluded by direct use of secondary antibody without adding primary antibody.

**Uncropped western blotting**

**Figure 1B**

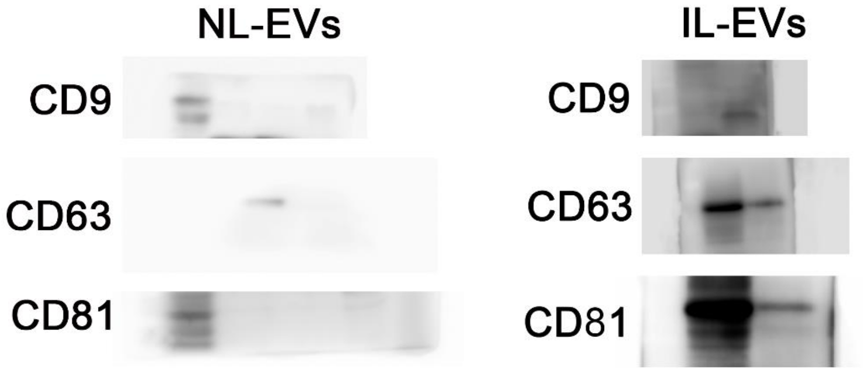


**Figure 5C**

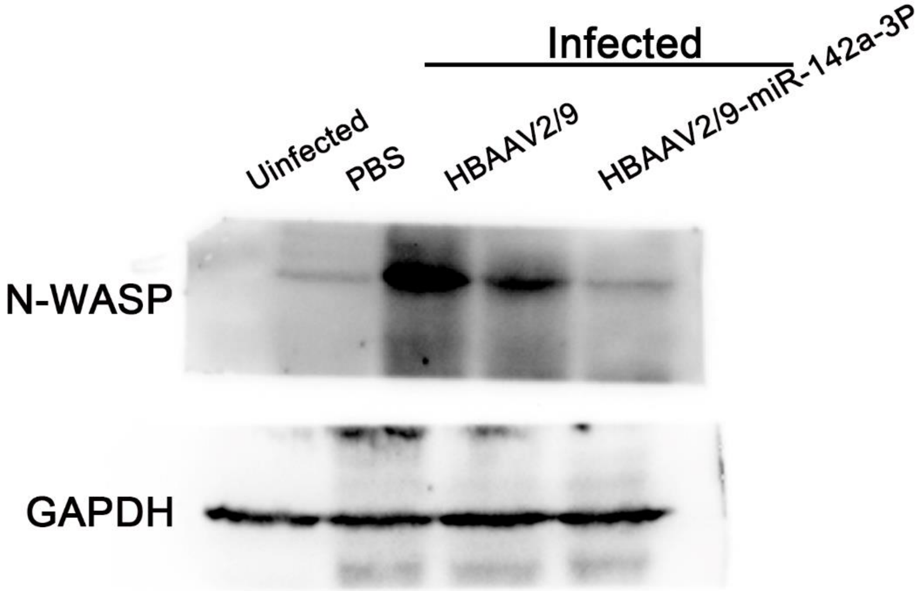


**Figure 7B**

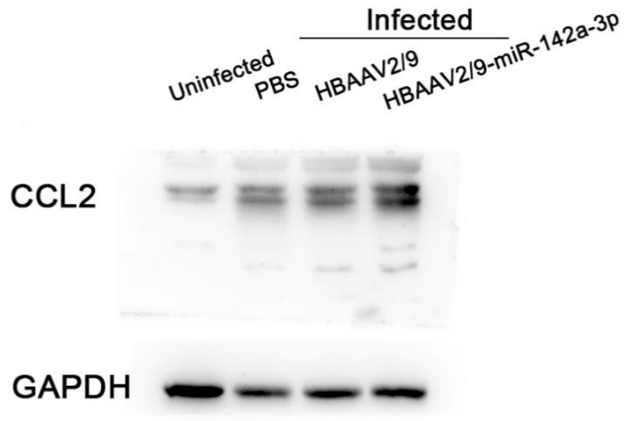


**Figure 7D**

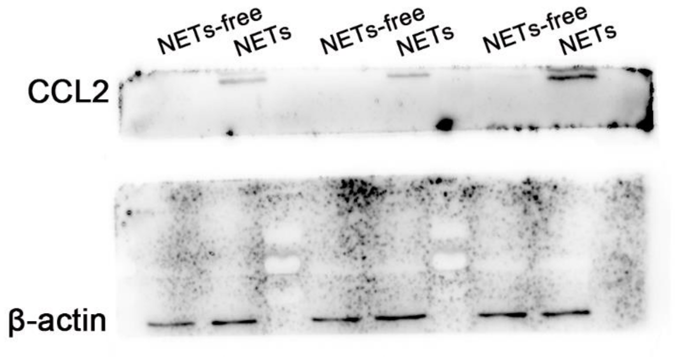


**Figure 8E**

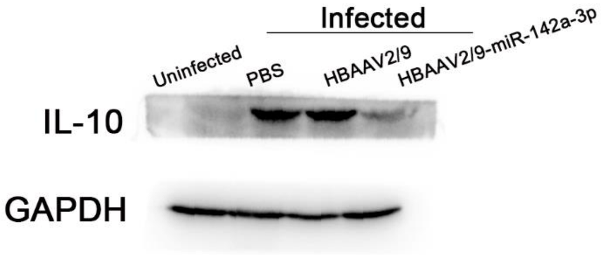


**Figure 8K**

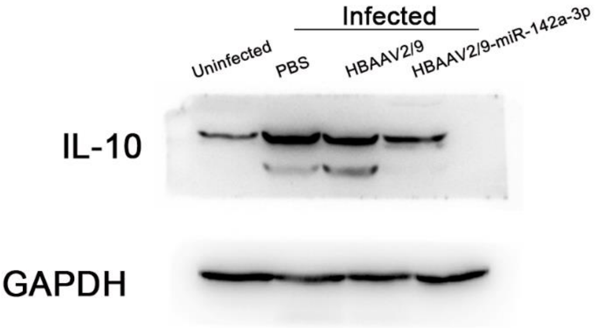


**Figure 8L**

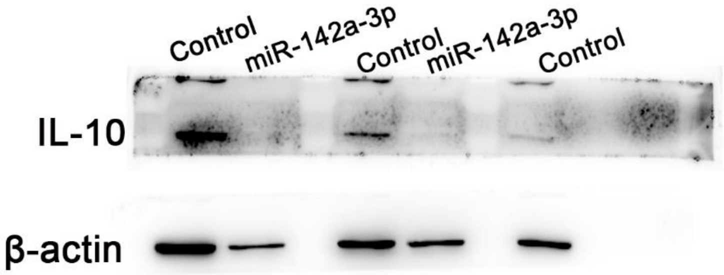


**Figure 8N**

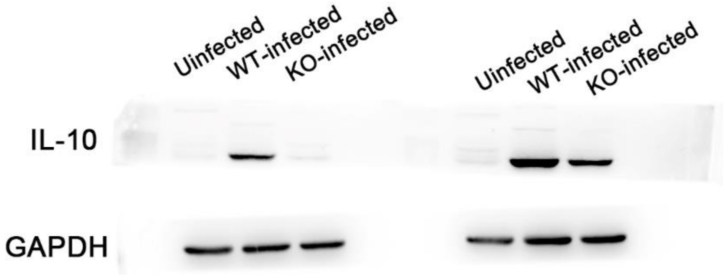

Supplement: Document S2. Article plus supplemental information [file mmc2.pdf]
